# ADME gene-driven prognostic model for bladder cancer: a breakthrough in predicting survival and personalized treatment

**DOI:** 10.1186/s41065-025-00409-4

**Published:** 2025-03-19

**Authors:** Haojie Dai, Xi Zhang, You Zhao, Jun Nie, Zhenyu Hang, Xin Huang, Hongxiang Ma, Li Wang, Zihao Li, Ming Wu, Jun Fan, Ke Jiang, Weiping Luo, Chao Qin

**Affiliations:** 1 The Affliated Liyang People’s Hospital of Kangda College of Nanjing Medical University, Changzhou, Jiangsu China; 2 The First Clinical Medical College, Nanjing Medical University, Nanjing, Jiangsu China; 3 Department of Urology, The First Affliated Hospital of Nanjing Medical University, Nanjing, China

**Keywords:** Bladder cancer, ADME genes, Prognostic prediction, Risk stratification model, CYP2C8

## Abstract

**Background:**

Genes that participate in the absorption, distribution, metabolism, excretion (ADME) processes occupy a central role in pharmacokinetics. Meanwhile, variability in clinical outcomes and responses to treatment is notable in bladder cancer (BLCA).

**Methods:**

Our study utilized expansive datasets from TCGA and the GEO to explore prognostic factors in bladder cancer. Utilizing both univariate Cox regression and the lasso regression techniques, we identified ADME genes critical for patient outcomes. Utilizing genes identified in our study, a model for assessing risk was constructed. The evaluation of this model's predictive precision was conducted using Kaplan–Meier survival curves and assessments based on ROC curves. Furthermore, we devised a predictive nomogram, offering a straightforward visualization of crucial prognostic indicators. To explore the potential factors mediating the differences in outcomes between high and low risk groups, we performed comprehensive analyses including Gene Ontology (GO) and Kyoto Encyclopedia of Genes and Genomes (KEGG)-based enrichment analyses, immune infiltration variations, somatic mutation landscapes, and pharmacological sensitivity response assessment etc. Immediately following this, we selected core genes based on the PPI network and explored the prognostic potential of the core genes as well as immune modulation, and pathway activation. And the differential expression was verified by immunohistochemistry and qRT-PCR. Finally we explored the potential of the core genes as pan-cancer biomarkers.

**Results:**

Our efforts culminated in the establishment of a validated 17-gene ADME-centered risk prediction model, displaying remarkable predictive accuracy for BLCA prognosis. Through separate cox regression analyses, the importance of the model’s risk score in forecasting BLCA outcomes was substantiated. Furthermore, a novel nomogram incorporating clinical variables alongside the risk score was introduced. Comprehensive studies established a strong correlation between the risk score and several key indicators: patterns of immune cell infiltration, reactions to immunotherapy, landscape of somatic mutation and profiles of drug sensitivity. We screened the core prognostic gene CYP2C8, explored its role in tumor bioregulation and validated its upregulated expression in bladder cancer. Furthermore, we found that it can serve as a reliable biomarker for pan-cancer.

**Conclusion:**

The risk assessment model formulated in our research stands as a formidable instrument for forecasting BLCA prognosis, while also providing insights into the disease's progression mechanisms and guiding clinical decision-making strategies.

**Supplementary Information:**

The online version contains supplementary material available at 10.1186/s41065-025-00409-4.

## Introduction

Annually, bladder cancer (BLCA) accounts for about 3% of new global cancer diagnoses, ranking it as the 11th most common cancer. It is estimated that each year sees around 573,000 new diagnoses [1]. BLCA arises from a complex interplay of factors such as genetics, environmental and occupational influences, tobacco use, and conditions linked to obesity [2]. In terms of genetics, the notorious Lynch syndrome increases the risk of developing BLCA [3]. Smoking, especially black smoke, leads to BLCA mainly because of the high amount of carcinogens in it and is metabolized by the kidneys and enriched in the bladder [4]. As for occupational exposure, it has been reported that the rate of BLCA is significantly higher in workers who are chronically exposed to aromatic amines, PAH, such as petroleum workers, metal factory workers [5]. Up to 2024, the 5-year survival rate of BLCA is encouraging, nearing 78%, suggesting a favorable prognosis, but is the fourth leading cause of cancer deaths in men over 80 years of age [6]. Nevertheless, metastatic BLCA presents a sharply reduced overall survival (OS) rate, which dips under 6% [7]. Significantly, around half of those initially diagnosed with muscle-invasive BLCA manifest recurrences and progress to metastasis [8]. In addition, the extensive exploration of cancer biology since the past centuries has facilitated the implementation of precision treatments, such as integrating surgery and chemotherapy, selecting appropriate molecular drugs, and breaking through the barriers of the immune microenvironment [9]. In summary, the malignant evolution of bladder cancer and the progress in precision cancer therapy emphasize the urgent need to construct reliable biomarker models.

The drug ADME (absorption, distribution, metabolism, excretion) gene superfamily is instrumental in the intricate mechanisms of drug absorption, distribution, metabolism, and elimination [10–12]. Per the PharmaADME consortium (http://www.pharmaadme.org), the superfamily encompasses 266 genes, including a fundamental subset of 32 genes classified by their specific functions in pharmacokinetics including drug transporters, regulatory factors, and enzymes active in phase I and II drug metabolism [13–15]. In addition to affecting individual drug response [16], polymorphisms of ADME genes are closely associated with many aspects of cancer. For example, single nucleotide polymorphisms in the metabolic gene AKR1C3 have been associated with bladder cancer [17] and leukemia [18], polymorphisms in genes such as CYP17A1 affect drug efficacy in prostate cancer [19], and 3'UTR alterations in a group of ADME genes promote the risk of toxicity in breast cancer treatment [20]. Moreover, significant intergroup variations exist in the transcriptional, translational, and epigenetic modulation of ADME gene expression [21–23].

Recent studies have provided insights into the prognostic potential of ADME heterogeneity in renal cancer from the perspective of spatial transcriptome combined with single-cell data as well as bulk RNA-seq, deepening the understanding of the regulatory role of ADME genes in the tumor microenvironment [24, 25]. Hu and associates have pinpointed critical ADME genes that predict overall survival in a range of cancers [26]. Research by Han et al. revealed connections between ADME gene variations and survival outcomes in non-small cell lung carcinoma [27]. Tang and his team have associated these genes with prognostic results and the extent of immune cell infiltration in head and neck squamous cell carcinoma [28].

Although progress has been made in understanding the function of ADME genes in cancer, the potential clinical significance of genetic anomalies related to ADME in BLCA has not been fully explored. In this research endeavor, we sought to unravel the molecular alterations and clinical significance of ADME-associated genes in BLCA. We introduced a novel risk prediction model to evaluate treatment outcomes and prognosis in BLCA patients. Independent cohorts were used to rigorously validate this model, confirming its strong predictive capabilities and its potential to influence clinical decisions. The deployment of this model could imminently facilitate the development of customized treatment strategies for patients diagnosed with BLCA.

## Materials and methods

### Data collection

Clinical information and transcriptional profiles from the TCGA-BLCA and TCGA-Pan-cancer dataset were retrieved through the UCSC Xena platform (https://xena.ucsc.edu) [29]. During our analysis, any subjects missing complete survival data or RNA sequencing details were systematically excluded. We obtained the GSE13507 gene expression dataset [30] as independent validation set, which includes 165 primary bladder cancer specimens, from the Gene Expression Omnibus (GEO) repository. The GSE13507 cohort was widely used as a validation set for BLCA prognostic models [31–33]. Data from the IMvigor 210 cohort, detailing clinical and characteristic information, is accessible through the 'IMvigor210CoreBiologies' R package. 298 ADME genes were obtained from the supplementary table in the previous literature of Tang et al. [28].

### Development and validation of a prognostic risk prediction model using ADME genes

In our study of the TCGA-BLCA cohort, based on the threshold selection strategy in previous literature [24, 34], by setting a log2FoldChange threshold greater than 1 and an adjusted *P*-value under 0.05, researchers identified genes exhibiting differential expression between tumor tissues and adjacent normal tissues through “edgeR” R package. To evaluate their prognostic significance, a univariate Cox regression analysis was performed on ADME-related differentially expressed genes (DEGs) [34]. Lasso regression is known for its efficiency in screening important features and can effectively deal with the problem of multicollinearity of parameters, avoiding overfitting to a great extent [35]. In our study, a robust risk assessment model was developed by identifying the optimal regularization parameter, lambda (λ), through Lasso cox regression analysis paired with tenfold cross-validation. This approach ensured precise determination of λ, enhancing the model’s efficacy. Risk scores for patients were derived by integrating standardized gene expression values (Expi) with their respective regression coefficients (Coei), following a strictly defined algorithmic framework:

$$Risk score=\sum_{\text{i}=1}^{\text{n}}(Expi \times Coei)$$

Risk categorization of samples into high-risk and low-risk groups was performed using the median risk score as the dividing criterion. Drawing on the analytic strategies of previous studies, the disparities in survival between the categories were assessed through Kaplan–Meier survival curves and analyzed using log-rank tests [36–38]. To confirm the predictive accuracy of our risk model, time-dependent ROC curves were constructed [39, 40]. In addition, Decision Curves Analysis (DCA) was performed to aid in the understanding of the prognostic potential of the model [38]. The robustness of our model's predictive capability was further validated using BLCA samples sourced from the GEO database, emphasizing its consistent performance across diverse datasets, thereby enhancing its credibility and dependability.

### Establishment and validation of the nomogram

Cox proportional hazards regression analysis was utilized to ascertain the independent prognostic capability of the risk score. A nomogram was formulated, integrating key clinical parameters—age, gender, pathological stage, and the risk score—to predict outcomes, aimed at refining the precision of prognostic predictions [40, 41]. The predictive accuracy of this nomogram was rigorously scrutinized using calibration curves, DCA curves and ROC analysis, underscoring its reliability and clinical applicability in predicting patient outcomes.

### Enrichment analysis in high- and low-risk groups

By setting a log2FoldChange threshold above 1 and keeping the adjusted *P*-value below 0.05, we pinpointed genes that were differentially expressed between high- and low-risk groups. These DEGs underwent extensive functional characterization using the GO and KEGG databases to unravel their biological significance. Furthermore, we performed Gene Set Enrichment Analysis (GSEA) tailored to GO and KEGG datasets, highlighting unique metabolic pathways enriched in each risk group, providing deeper insights into the molecular underpinnings of their distinct risk profiles. The results of enrichment analysis are presented according to adjusted *P*-value.

### Immune infiltration analysis

Using the 'ESTIMATE' R package, we derived stromal, immune, and overall ESTIMATE scores to delineate the cellular composition of the tumor microenvironment (TME) in each specimen. To characterize the TME in BLCA patients, using the CIBERSORT algorithm [42] in conjunction with ssGSEA methods, the extent of immune cell infiltration was quantified. Following quantification, Spearman correlation analysis was utilized to investigate the association between immune cell infiltration and the risk score. In addition, we analyzed the differences in the expression of human leukocyte antigen (HLA) family members and immune function assessed by ssGSEA between high and low risk subgroups.Finally we uncovered correlations between immune checkpoint expression and risk scores and explored the potential of risk scores as a prognostic indicator for immunotherapy in the cohort Imvigor210 treated with anti-PD-L1.

### Malignancy analysis, somatic mutation analysis and evaluation of chemotherapeutic drug sensitivity

By ssGSEA, we assessed the Angiogenesis score as well as the epithelial-mesenchymal transition (EMT) score for each sample. By applying the “maftools” R package, we carefully analyzed the data on somatic mutations and compared tumor mutation burden (TMB) in different risk subgroups. KM curves were used to demonstrate survival differences between subgroups. We utilized the 'oncoPredict' R package to evaluate differential drug responsiveness across high- and low-risk cohorts, revealing potential therapeutic approaches informed by our findings.

### Construction of ADME-related clusters

“ConsensusClusterPlus” R package was utilized to identify diverse ADME patterns and cluster patients in TCGA cohort into multiple categories for further study. Principal component analysis (PCA) and tSNE were applied to distinguish clustered differences in expression profiles. KM curves were drawn to demonstrate the prognostic characteristics of different clusters. In addition, we explored differences in pathway activation across clusters by GSEA and GSVA.

### Identification of core gene

Utilizing the STRING database (http://string-db.org), we constructed a PPI network from genes we identified as prognostic markers.The protein that communicates most extensively with other proteins was identified as the core of the network. Core gene-associated ppi networks were constructed with GeneMANIA (https://genemania.org/). We assessed the prognostic potential of the core genes by KM curves and revealed possible means of regulation by immune infiltration assessment as well as GSVA analysis. In addition, we screened for differential genes between high and low expression groups by the same criteria as before, and based on this we performed KEGG enrichment analysis to deepen our understanding of the mechanism.

### Cell culture

Four BLCA cell lines (T24, UMUC3, RT4, and BIU87) and normal cells (SVHUC) were obtained from the Cell Bank of the Chinese Academy of Sciences (Shanghai, China). BIU87 cells were treated with 10% PMI-1640 medium, T24 cells with 10%McCoy's 5A medium, RT4 cells with 10% DMEM medium, and UM-UC-3 cells with 10% MEM medium. SV-HUC-1 was cultured using 10% Ham's F-12 K medium in a constant temperature incubator at 37 °C with 5% CO2 volume fraction using culture bottles. All were cultured at 37 °C in a humidified atmosphere containing 5% CO_2_.

### Quantitative real-time PCR (qRT-PCR) and immunohistochemical analysis

Our previous study described specific methods for total RNA extraction and qRT-PCR. The following primers were used in this study:

- CYP2C8 forward, 5'-CATTACTGACTTCCGTGCTACAT-3' and
- CYP2C8 reverse, 5'-CTCCTGCACAAATTCGTTTTCC-3';
- Actin forward, 5'-GAAGATCAAGATCATTGCTCCTC-3' and
- Actin reverse, 5'-ATCCACATCTGCTGGAAGG-3’.

Actin was used as an internal control. The procedures were performed three times to ensure accuracy and precision. The relative expression levels were calculated using the 2^−ΔΔCT^ method. Immunohistochemical images for the gene were sourced from The Human Protein Atlas, comparing both cancerous and non-cancerous bladder tissues.

### Core gene-based pan-cancer analysis

Pan-cancer differential expression plot of the core genes were downloaded from Timer website. Then by univariate cox analysis, we analyzed the potential of the core genes as predictors of OS, Progression Free Interval (PFI), and Disease Free Interval (DFI) in the TCGA-Pan-cancer dataset, and demonstrated the KM curves in a number of significantly discriminatory cancer types. Subsequently, we explored the correlation of core gene expression with immune checkpoint expression and abundance scores derived from the Cibersort algorithm.In addition, we explored the correlation between core gene expression and scores based on KEGG's immune-inflammation-related gene set after assessing the enrichment score by the ssGSEA method.

### Statistical analysis

To assess predictive accuracy, the analysis was conducted using ROC curves, utilizing the AUC as the key quantitative measure. Additionally, we employed the Wilcoxon test for nonparametric analysis. In analyzing the data, the software R, version 4.3.3, was employed. The significance of differences among groups was determined with a threshold *P*-value set at below 0.05, while the significance test for DEGs was then based on an adjusted *P*-value less than 0.05. *P*-value correction was done by the Benjamini-Hochberg (BH) method.

## Results

### Screening of differentially expressed and prognosis-associated ADME genes in BLCA

**Fig. 1:**
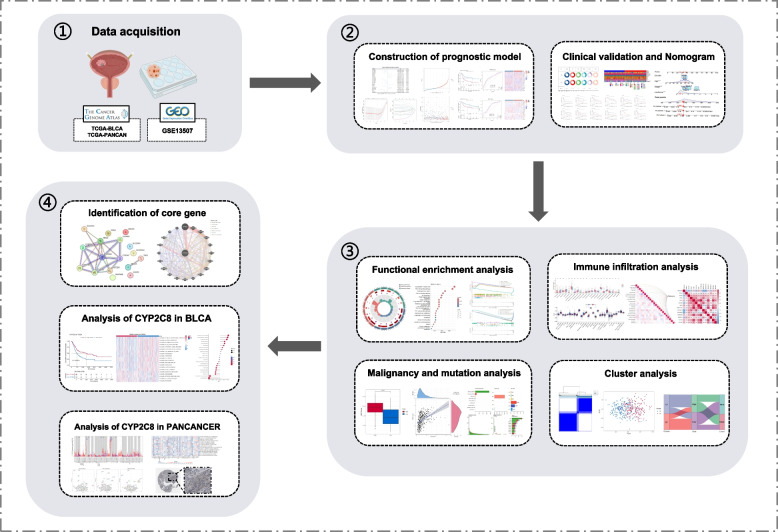
Flowchart of this study

**Fig. 2:**
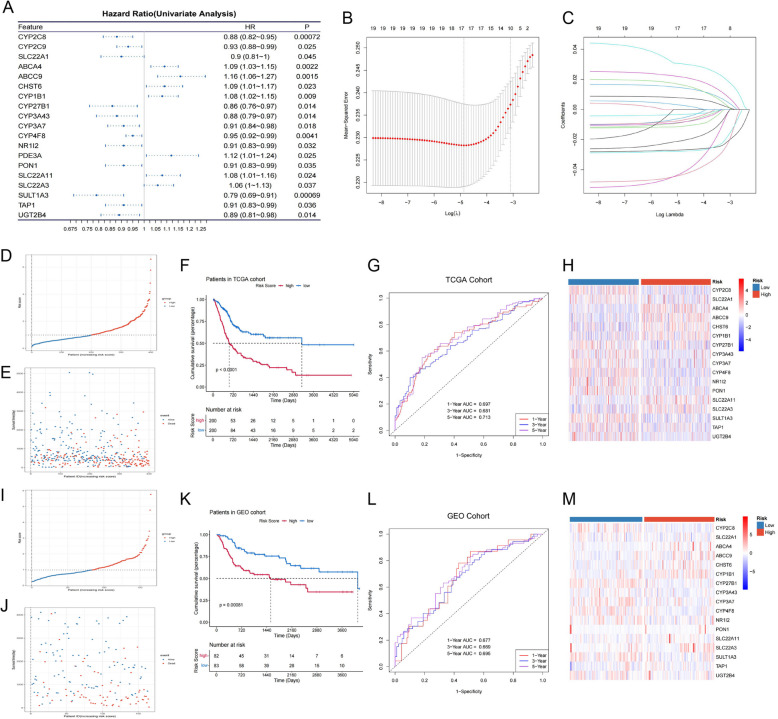
Construction of ADME-related BLCA risk model. **A** Forest plot capturing 19 ADME genes pinpointed through univariate Cox regression as potential prognostic indicators. **B** and** C** Lasso regression analysis reduces dimensions, focusing on these 19 genes. **D** and **I** Categorizing both the training cohort and the validation cohort into high-risk and low-risk groups based on risk scoring. **E** and** J** Scatter plots comparing survival time to survival outcomes within the training and validation cohorts. **F** and** K** Kaplan–Meier curves depicting OS for high- and low-risk groups in both cohorts. **G** and **L** The ROC curves for prognosis prediction based on risk scores in both cohorts. **H** and** M** Heatmaps illustrating the expression levels of prognosis-related ADME genes within high-risk and low-risk groups for both cohorts

The specific steps of the study are detailed in the flowchart (Fig. 1). Utilizing the RNA-seq data from the TCGA dataset, we meticulously analyzed the transcriptional profiles of BLCA and corresponding normal samples, uncovering a total of 6444 genes that displayed significant dysregulation (adjusted *P*-value < 0.05), including 80 ADME genes. The prognostic significance of specific ADME genes was assessed using univariate Cox regression analysis. This evaluation demonstrated that 19 of these genes significantly correlate with patient outcomes (Fig. 2 A). We applied Lasso regression to enhance the precision of the analysis, effectively reducing the list of prognostic ADME genes from 19 to 17 key genes associated with prognosis: CYP2C8, SLC22A1, ABCA4, ABCC9, CHST6, CYP1B1, CYP27B1, CYP3A43, CYP3A7, CYP4F8, NR1I2, PON1, SLC22A11, SLC22A3, SULT1A3, TAP1, and UGT2B4 (Fig. 2 B-C).

### Development and validation of a prognosis-related ADME gene risk prediction model

Utilizing multivariate Cox analysis, we constructed predictive models and obtained risk scores for each sample. In the TCGA training dataset for BLCA patients, we divided the subjects into high-risk and low-risk groups based on the median risk score (Fig. 2 D). The scatterplot visualized the increase in the number of dead patients and the decrease in survival time as the risk score increased (Fig. 2 E), while the Kaplan–Meier curves highlighted the significant OS differences between the high and low risk groups (Fig. 2 F). This certainly demonstrated the excellent prognostic stratification ability of the model. The ROC curves were used to evaluate our model's predictive accuracy (Fig. 2 G), revealing AUC scores of 0.697, 0.681, and 0.713 for the 1-, 3-, and 5-year overall survival forecasts, respectively. We visually details the expression patterns of 17 ADME genes associated with prognosis, displayed across two risk clusters in a heatmap format (Fig. 2 H). To fortify the credibility of our risk prediction model, we executed validation using an independent GEO dataset (GSE13507, *n* = 165). After distinguishing between high and low risk groups (Fig. 2 I), scatter plots (Fig. 2 J) alongside Kaplan–Meier curves (Fig. 2 K) from the test set illustrated significantly shorter overall survival times for patients in the high-risk group when compared to their low-risk counterparts. We then depicts the validation cohort's predictive outcomes, showcasing the AUC scores of 0.677, 0.669 and 0.695 for the 1-, 3-, and 5-year overall survival predictions, respectively (Fig. 2 L). Furthermore, to delineate the expression variations of the 17 ADME genes linked to prognosis across different risk levels, a heatmap was utilized (Fig. 2 M). In addition, we plotted DCA curves and calibration curves in the TCGA as well as the GEO cohort (Fig S1), and the high sensitivity further reflected the potential of the model to guide the process of clinical application.

### Identification of clinical features of the ADME-related model

**Fig. 3:**
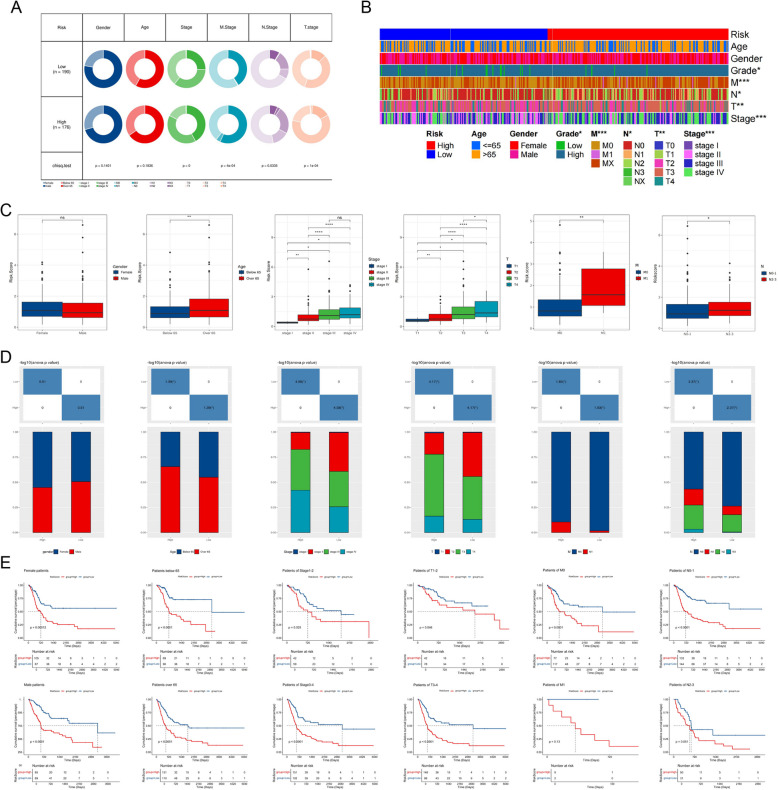
Association between ADME-related signature and clinical features. **A** and** B** Pie chart and heatmap showing the distribution of people with different clinical characteristics in the high and low risk groups. **C** Comparison of ADME-related score in different clinical groups. **D** Proportional differences in clinical characteristics between high- and low-risk groups. **E** Validating the prognostic accuracy of our model in cohorts with different clinical characteristics

Differences in the distribution of clinical characteristics in high- and low-risk subgroups were exquisitely illustrated in pie charts and heatmap (Fig. 3A-B). Initially, we compared risk scores in cohorts of patients with different clinical characteristics. It was found that although risk scores did not differ significantly between male and female genders, patients of advanced age and those with advanced pathologic staging demonstrated more significantly higher risk scores (Fig. 3 C). In addition, we analyzed the distribution of clinical characteristics in different risk subgroups (Fig. 3 D). Interestingly, there was a significantly higher proportion of patients with advanced pathologic stage as well as advanced age in the high-risk group. Subsequent KM survival analyses demonstrated the power of ADME-related model to differentiate patient survival across diverse cohorts, and we found that highly scored patients survived worse across patients with different clinical characteristics (Fig. 3 E). These results indicate a significant negative correlation between ADME-related score and prognosis in BLCA patients, which further validated the prognostic accuracy of our model.

### Prognostic significance of the risk score

**Fig. 4:**
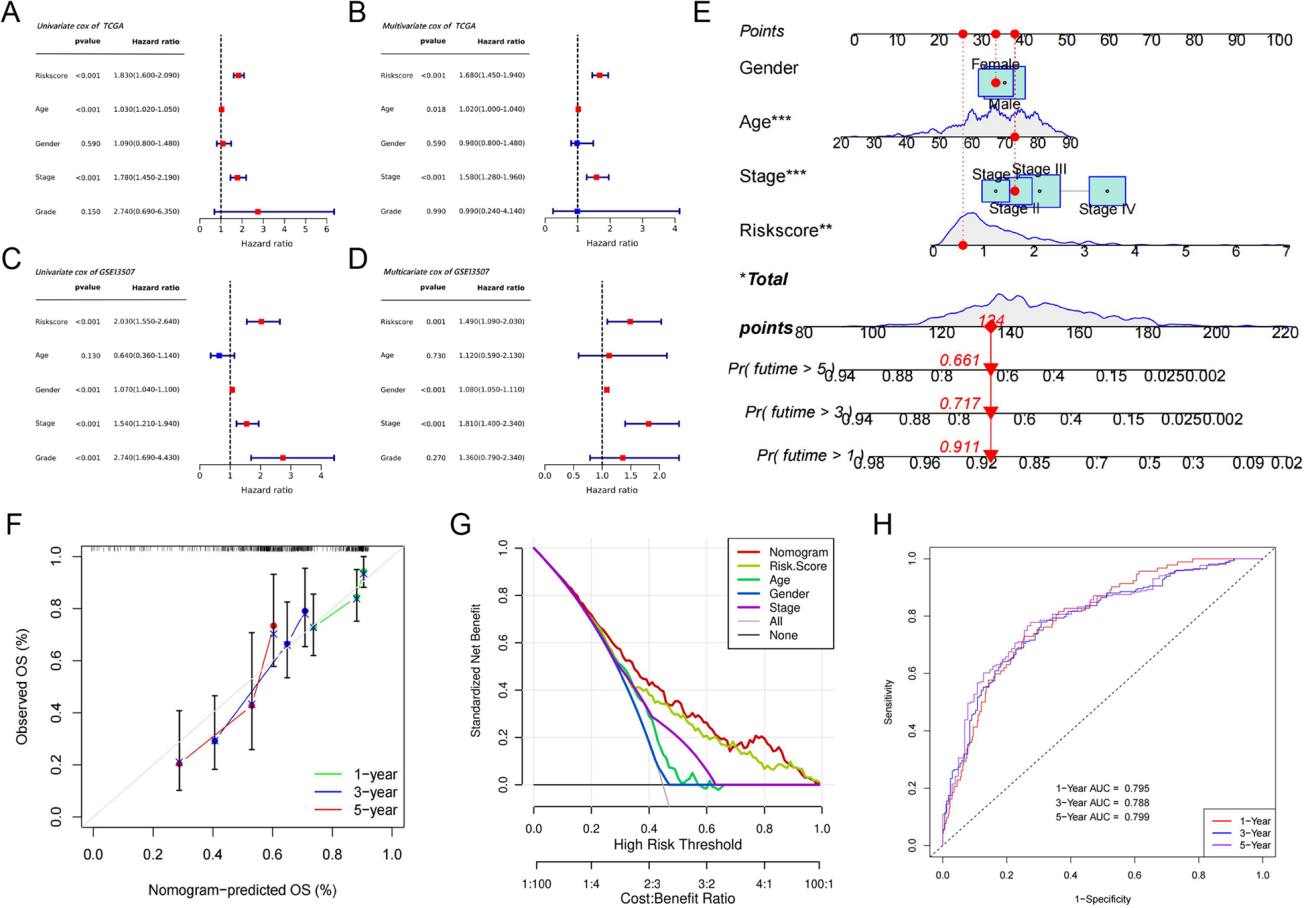
Risk score as an independent prognostic indicator for BLCA patients, alongside the development and confirmation of a Nomogram. **A** and **C** Forest plots from univariate Cox regression analysis within the TCGA and GEO cohorts. **B** and **D** Forest plots from multivariate Cox regression analysis for both cohorts. **E** A Nomogram designed to forecast OS rates in BLCA patients. **F** Calibration curves associated with the Nomogram. **G** DCA curves associated with Nomogram features. **H** The ROC curves evaluating the Nomogram's predictive performance

To assess the risk score's effectiveness as an independent predictor for BLCA, both univariate and multivariate Cox regression analyses were performed. Statistical analysis revealed that, within the training cohort, the risk score significantly influenced prognosis (Fig. 4 A and B). This finding was subsequently corroborated in an external cohort, reinforcing the reliability of the risk score as a prognostic biomarker (Fig. 4 C and D). To refine prognostic precision, we merged the training set with the validation set to construct a nomogram, incorporating the risk score with variables such as gender, age, and stage (Fig. 4 E). The model's predictive precision was demonstrated by a calibration plot that matched the ideal trajectory and DCA curves (Fig. 4 F and G). The evaluation of the nomogram through ROC analysis produced AUC values of 0.795, 0.788, and 0.799 for predictions of 1-, 3-, and 5-year overall survival, respectively (Fig. 4 H), which underscores the nomogram’s robust predictive capability.

### Functional enrichment analysis

**Fig. 5:**
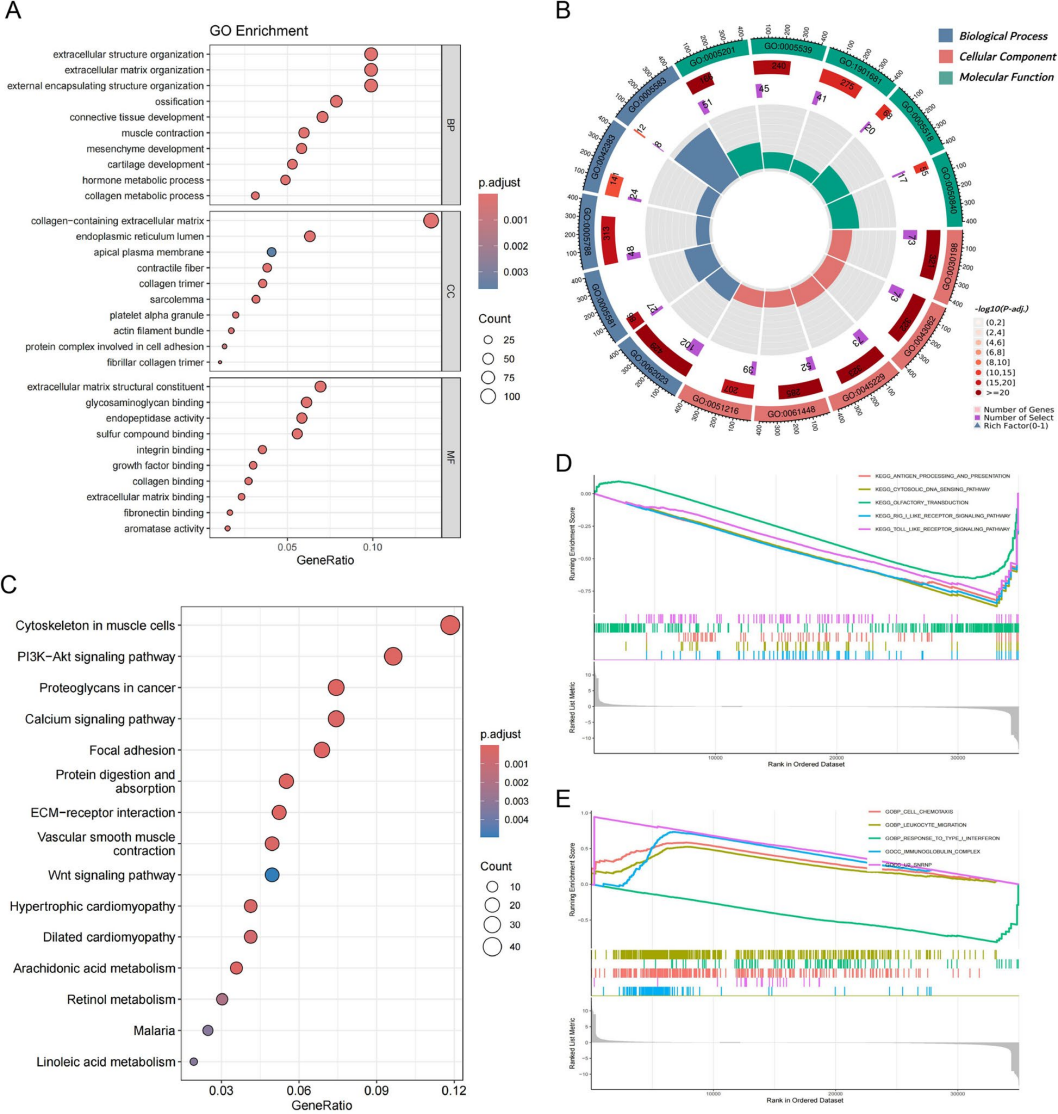
Enrichment analyses for high- and low-risk categories. **A** and** B** The GO enrichment assessments of DEGs. **C** The KEGG analysis. **D** and **E** GSEA between the two groups

In-depth investigations leveraged GO and KEGG databases to elucidate the functional distinctions of genes with varying expression profiles across high- and low-risk cohorts. GO analysis identified significant clustering in biological functions, specifically concerning the organization of the extracellular matrix and associated functions. Key areas include the architecture of collagen-rich extracellular matrices, activation of signaling receptors, and interactions between receptors and ligands (Fig. 5 A-B). Moreover, the KEGG analysis illuminated the enrichment of DEGs within a series of cancer-promoting pathways including PI3K − Akt signaling pathway, Proteoglycans in cancer and Focal adhesion (Fig. 5 C). To delve deeply into the functional pathways, GSEA was also utilized. In this analysis, immune-related pathways, particularly those involving response to type-I interferon, antigen processing and presentation, toll-like receptor signaling cascades showed a significantly higher prevalence in the low-risk group (Fig. 5 D). We observed significant enrichment of pathways concerning immune function, notably leukocyte migration and immunoglobulin complex activities, in the high-risk group (Fig. 5 E). These distinctions outline unique immune signatures for the two groups.

### Immune infiltration analysis

**Fig. 6:**
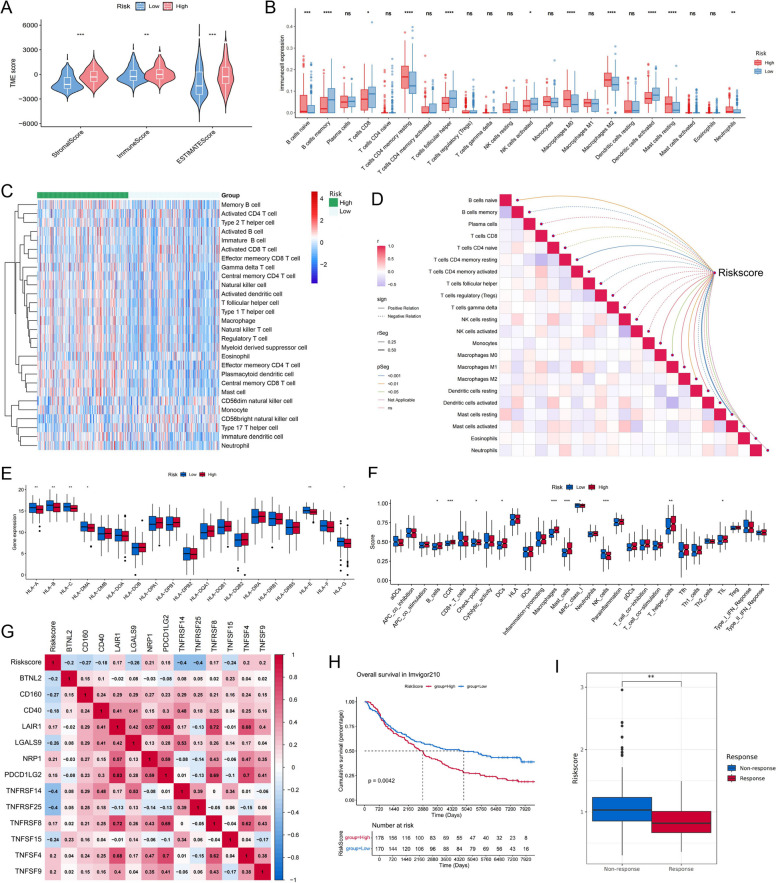
Immune infiltration analyses and immunotherapeutic analyses. **A** The TME scores contrasting low and high-risk patients. **B** Boxplots of the infiltration levels of 22 specific immune cells. **C** Heatmap visually differentiating the quantities of 28 immune cells between high- and low-risk groups. **D** The relationships between risk scores and the infiltration of immune cells. **E** Differential expression of HLA-related genes. **F** Landscape of immune function differences. **G** The correlation between risk score and immune checkpoints, assessed using the Pearson test, where a significance of *P* < 0.001 was recorded. **H** The Kaplan–Meier curve comparing OS between the two risk subgroups in IMvigor210 cohort. **I** Box plots examining the relationship between risk score and the effectiveness of immunotherapy

The above studies established a robust link between the ADME-associated risk score and immunological pathways, we therefore performed careful analyses of the tumor immune microenvironment. The high-risk subset was characterized by increased stromal, immune, and ESTIMATE scores (Fig. 6 A). Utilizing the cibersort algorithm enabled the evaluation of diverse immune cell subsets in the TCGA training cohort, enhancing the understanding of the tumor's immune landscape. We presented a boxplot that displayed the infiltration levels of multiple immune cells (Fig. 6 B). Notably, there were elevated concentrations of CD8 + T lymphocytes, in the low-risk group, the higher presence of activated dendritic cells and ollicular helper T cells were noted as well. Conversely, the high-risk group exhibited increased levels of naive B cells, quiescent CD4 + memory T cells, and both M0 and M2 macrophages, as well as resting mast cells. Utilizing the ssGSEA approach, the prevalence of various immune cell types was quantified and visually represented through a heatmap (Fig. 6 C). Spearman's correlation analyses emphasized significant linkages between the risk score and patterns of immune cell infiltration (Fig. 6 D). Moreover, we present the correlation between model genes and the abundance of immune cell infiltration in Fig S2. Interestingly, we found that the expression of many important HLAs was down-regulated in high-risk subgroups, such as HLA-A, B, and C (Fig. 6 E). Subsequently, we revealed differences in immune function between high- and low-risk subgroups, with MHC I-associated functions being more active in the low-risk subgroup and macrophage-associated functions being significantly up-regulated in the high-risk subgroup (Fig. 6 F). Moreover, we illustrates a strong inverse correlation between the risk score and the expression levels of various immune checkpoint genes, such as CD40, TNFRSF14 and TNFRSF25 (Fig. 6 G). The discovery of distinctive infiltrations of immune cells in subsets with varying risk levels led to a deeper analysis of the IMvigor 210 cohort, focusing on exploring the relationship between ADME scores and the efficacy of immunotherapy treatments. Findings indicated that individuals in the low-risk subset demonstrated not only better prognosis (Fig. 6 H), but also enhanced responses to immunotherapy (Fig. 6 I), suggesting a higher likelihood of benefit from immunotherapy in this subgroup.

### Malignancy as well as mutation analysis

**Fig. 7:**
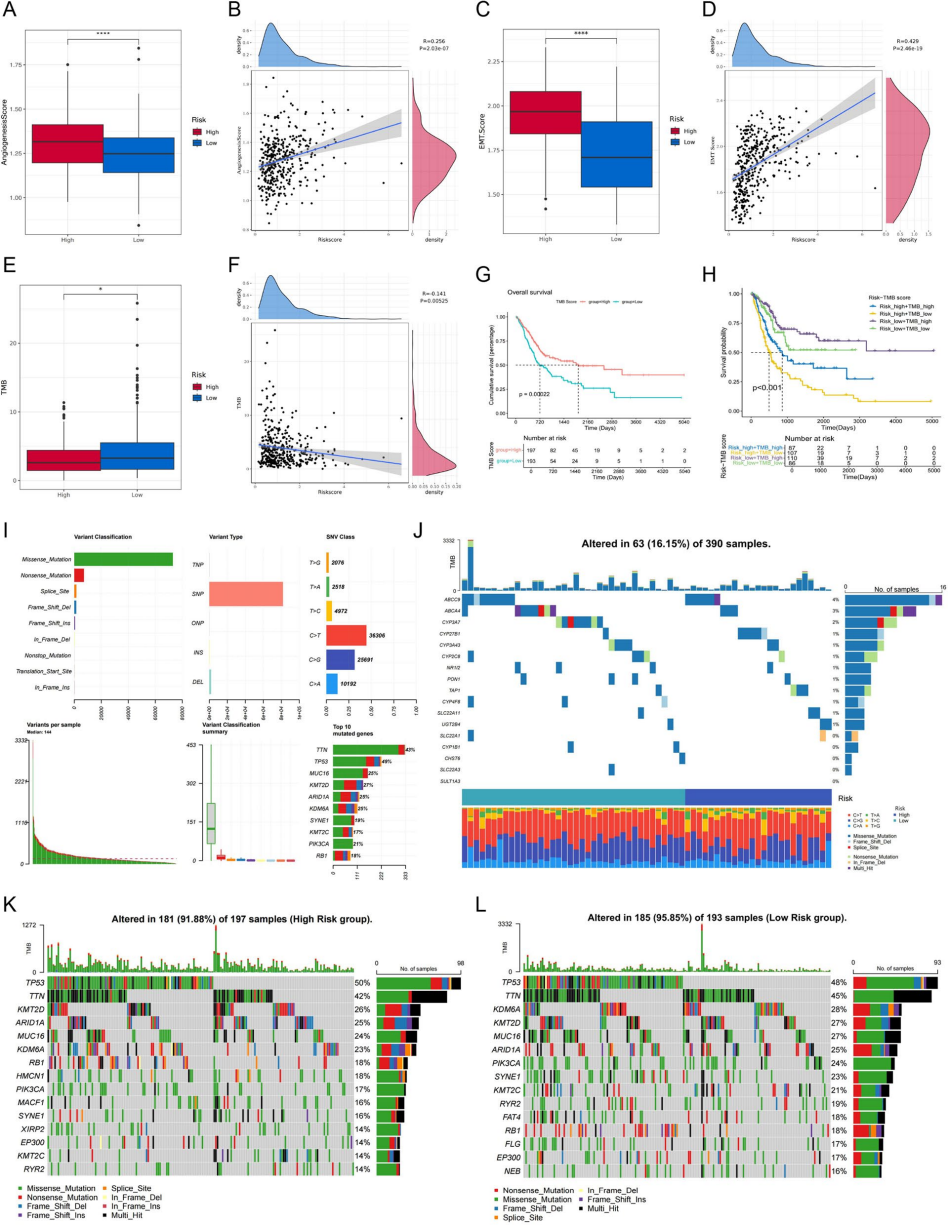
Malignancy and mutation analyses. **A** The difference of angiogenesis score in the two risk subgroups.** B** Correlation between angiogenesis score and risk score. **C** The difference of EMT score in the two risk subgroups.** D** Correlation between EMT score and risk score. **E** The difference of TMB in the two risk subgroups. **F** Correlation between TMB and risk score. **G** Survival curves of the two TMB ssubgroups. **H** Survival analysis between different subgroups classified by ADME-related risk score and TMB. **I** Overall mutation landscape of BLCA patients. **J** Waterfall plot demonstrates the mutation landscape of 17 prognostic genes in tumor samples. **K-L** Waterfall plots show the somatic mutations in the high- and low-risk subgroups, respectively

Initially, we analyzed the angiogenesis scores obtained based on ssGSEA as well as the EMT scores. The results showed that both were significantly upregulated in the high-risk group and had an extremely significant positive correlation with the risk score (Fig.7 A-D). Subsequently, we assessed differences in TMB across risk groups. Interestingly, we found that high TMB was enriched in the low risk group (Fig. 7 E) and revealed a negative correlation between TMB and risk scores (Fig. 7 F). Further KM analysis demonstrated good prognostic discriminatory validity of the TMB (Fig. 7 G), leading us to analyze the TMB in conjunction with ADME-related score. As we expected, the joint analysis demonstrated strong prognostic discrimination, indicating that patients with high risk and low TMB exhibited the worst prognosis, while those with low risk and high TMB showed the best prognosis (Fig. 7 H). In addition, we present the mutational landscape of the whole sample (Fig. 7 I). Moreover, we present not only the mutations of the 17 prognostic ADME genes (Fig. 7 J), but also the mutation ranking of the genes in the high and low subgroups in exquisite waterfall plots (Fig. 7 K-L). Interestingly, the pathway with the highest mutation rate was statistically found to be consistent in both the high and low risk subgroups, both being the RTK-RAS pathway. In addition, the low-risk group had a higher mutation rate of the WNT pathway, whereas the mutation rate of the Notch pathway in the high-risk group was much higher than that in the low-risk group (Fig. S3).

### Evaluation of medication reactions

**Fig. 8:**
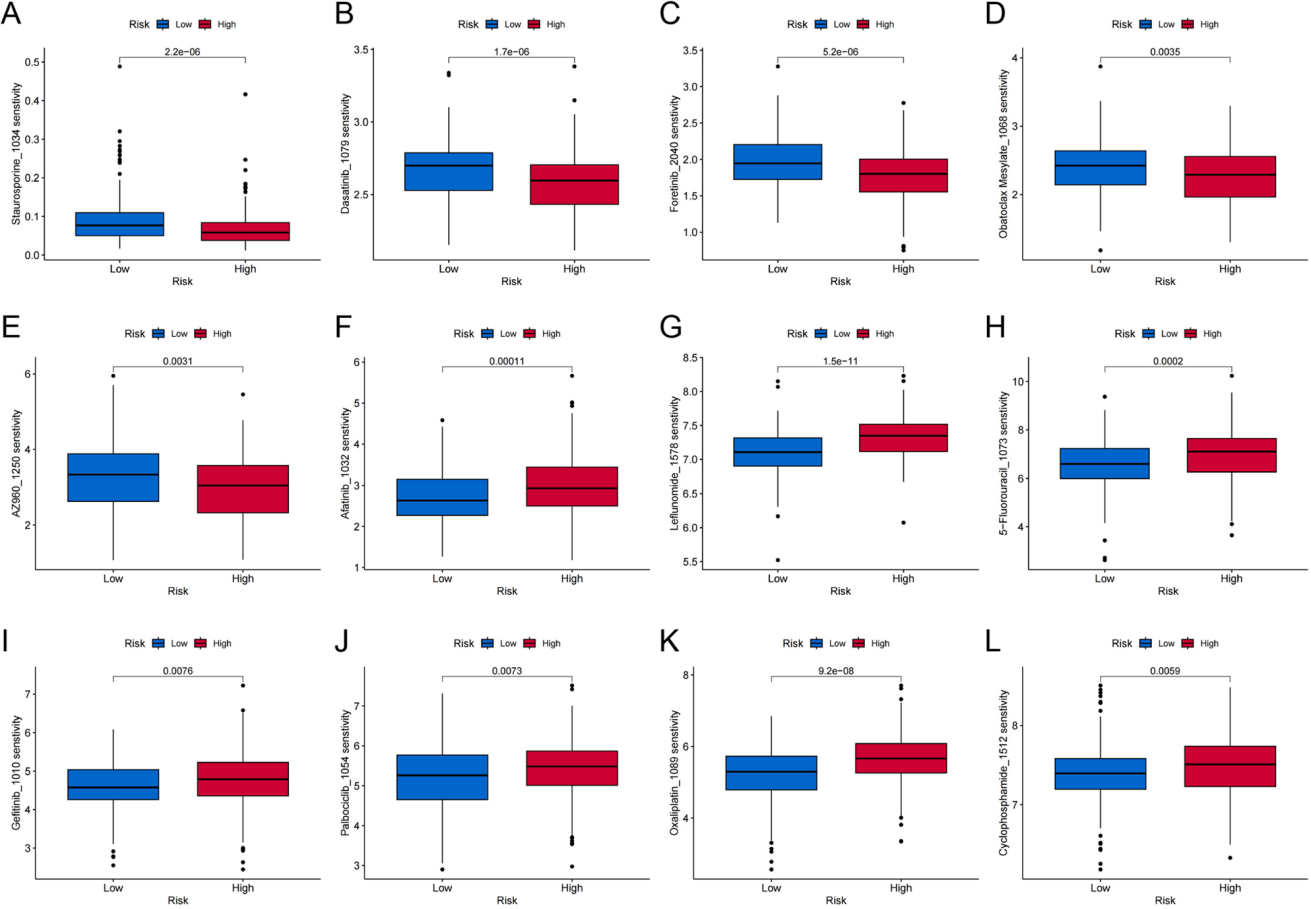
Box plots contrasts the responses to chemotherapy across two patient cohorts categorized by risk score

Between the cohorts classified as high- and low-risk, we then examined the variations in drug response. As illustrated in Fig. 8, the high-risk subset demonstrated reduced IC50 values for Staurosporine, Obatoclax Mesylate, Foretinib, Dasatinib, and AZ960 compared to the low-risk subset, suggesting an enhanced sensitivity to these medications. In contrast, the low-risk cohort displayed markedly reduced IC50 values for therapeutic agents such as Gefitinib, Afatinib, Oxaliplatin, Palbociclib, Leflunomide, and Cyclophosphamide, suggesting increased sensitivity to these drugs within this group. Comprehending the sensitivity profiles of both immunotherapeutics and chemotherapeutics provides crucial information that can facilitate the customization of treatment strategies tailored to individual patient characteristics.

### Cluster analysis based on prognostic genes

**Fig. 9:**
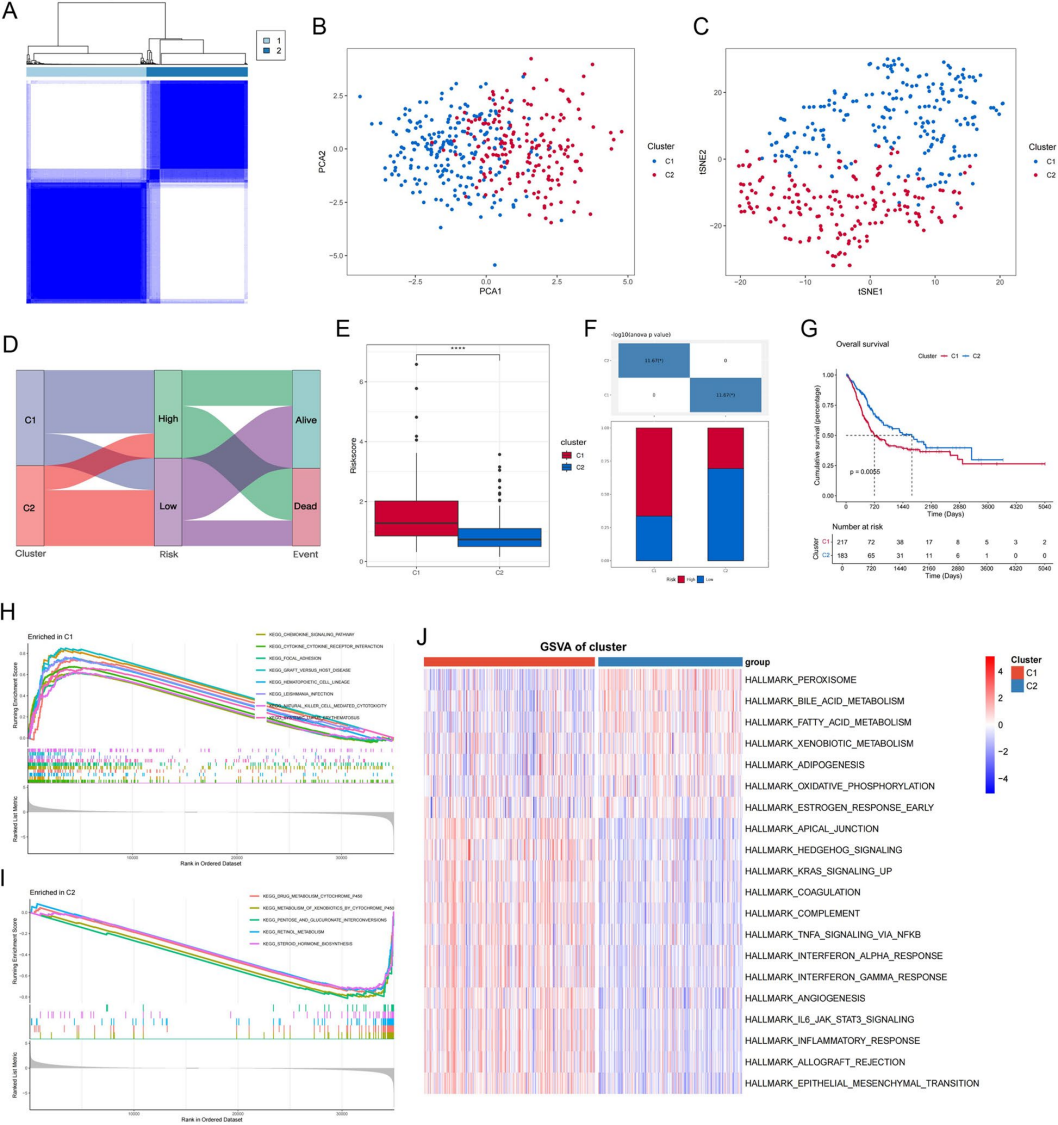
Analyses of ADME-related clusters. **A** Different ADME-related clusters were identified for k = 2 in TCGA cohort. **B** PCA analysis of the two clusters. **C** tSNE analysis of the two clusters (**D**) Sankey plot of clusters, risk and outcome. **E** The difference of risk score in two clusters. ****P < 0.0001. **F** Proportional differences of risk in different clusters. **G** Overall survival difference between clusters. **H-I** GSEA analysis in different clusters.** J** Heatmap of GSVA

Based on the expression of the 17 ADME genes, we clustered the TCGA samples into two categories for further exploration of the guidance potential of prognostic genes (Fig. 9 A). Plots of PCA and tSNE demonstrates that clustering has a good discriminatory effect on expression profiles (Fig. 9 B-C). Interestingly, the sankey diagram demonstrated that the C1 subgroup was pointed more toward high risk (Fig. 9 D). We then found that the C1 cluster exhibited higher risk scores (Fig. 9 E–F) and had a significantly lower prognosis than the other cluster (Fig. 9 G), corroborating the prognostic potential of the 17 ADME genes. In view of this, we tried to explore the pathway activation in different clusters. KEGG-based GSEA showed that pathways such as focal adhesion and cytokine-cytokine receptor interaction were significantly enriched in the C1 cluster, whereas the C2 cluster showed more intense metabolic dysregulation (Fig. 9 H-I). In addition, Hallmark-based GSVA heatmaps demonstrated significant upregulation of various pro-oncogenic pathways in the C1 cluster (Fig. 9 J).

### Identification and analysis of core gene

**Fig. 10:**
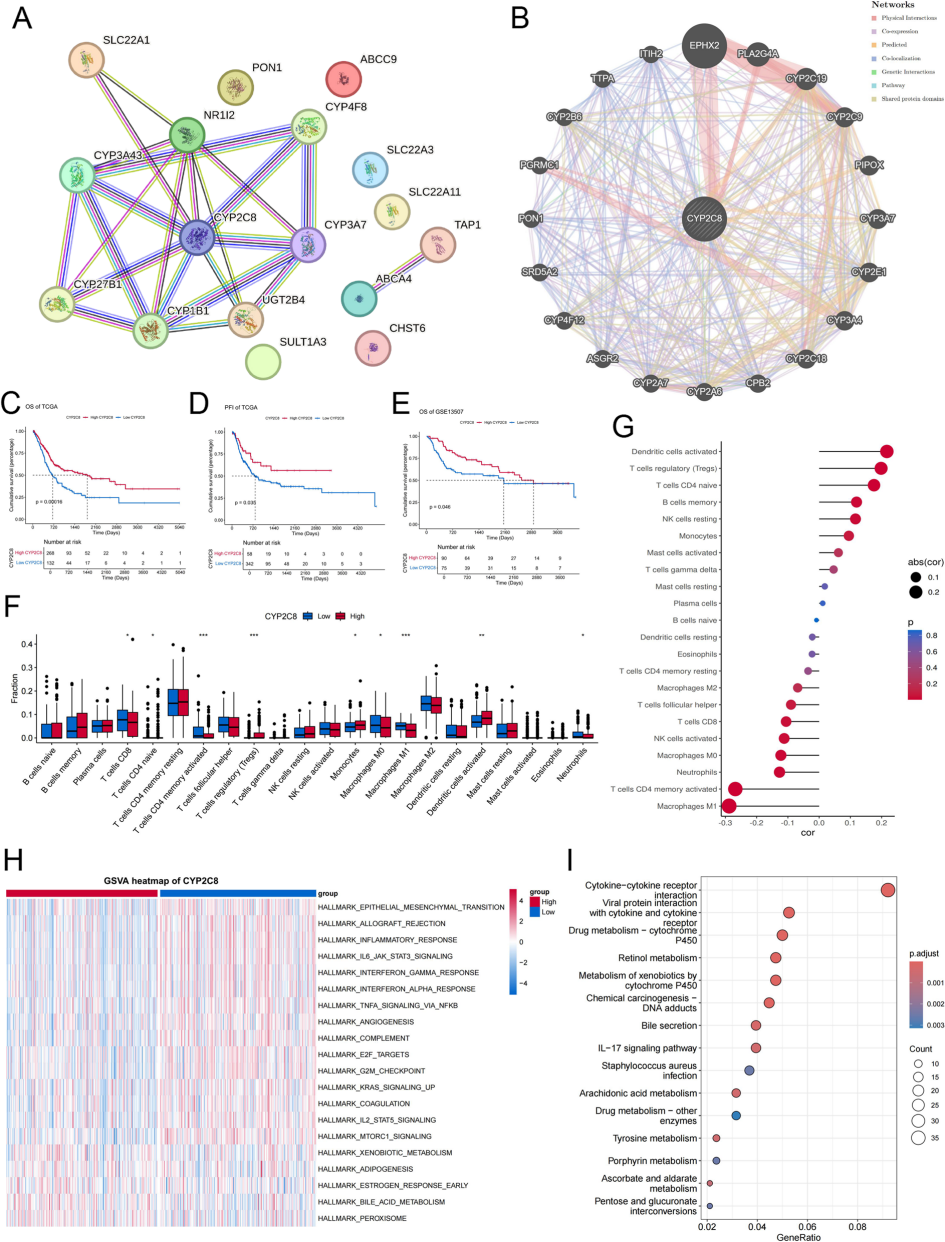
Identification and analysis of core gene (**A**) PPI network of prognostic genes (**B**) PPI network around CYP2C8 (**C**) OS segmentation of the TCGA cohort based on CYP2C8 expression (**D**) DFI segmentation of the TCGA cohort based on CYP2C8 expression (**E**) OS segmentation of the GSE13507 cohort based on CYP2C8 expression (**F**) Different immune landscapes in high and low expression groups **(G)** Correlation of CYP2C8 expression with immune cell abundance (**H**) Heatmap of GSVA in high and low expression groups (**I**) KEGG enrichment of DEGs between high and low expression groups

Firstly a PPI network was constructed from String database to investigate the functional relationships among 17 ADME genes associated with prognosis. This network illustrated complex interrelationships among the proteins involved (Fig. 10 A). Notably, CYP2C8 emerged as a central node within this network, suggesting its pivotal role. We then constructed a CYP2C8-centered PPI network through GeneMANIA, and interestingly, EPHX2 was the protein with which it most closely interacted (Fig. 10 B). Subsequent survival analyses showed CYP2C8 to be a reliable predictor of OS (Fig. 10 C) and PFI (Fig. 10 D) in the TCGA-BLCA cohort and were validated in the GSE13507 cohort (Fig. 10 E), where we found a better prognosis in the high expression group. Furthermore, we demonstrated different degrees of immune cell infiltration in the high and low CYP2C8 expression groups (Fig. 10 F) and analyzed the correlation between expression and infiltration abundance (Fig. 10 G). Interestingly, activated dendritic cells showed an extremely strong positive correlation with CYP2C8 expression. In addition, the heatmap of GSVA demonstrated significantly upregulated pro-oncogenic pathways in the low-expression group, which coincided with its poorer prognosis (Fig. 10 H). Subsequent KEGG enrichment based on differential genes between the high and low expression groups indicated that cytokine-related pathways as well as metabolic pathways were significantly dysregulated between the two groups (Fig. 10 I).

**Fig. 11:**
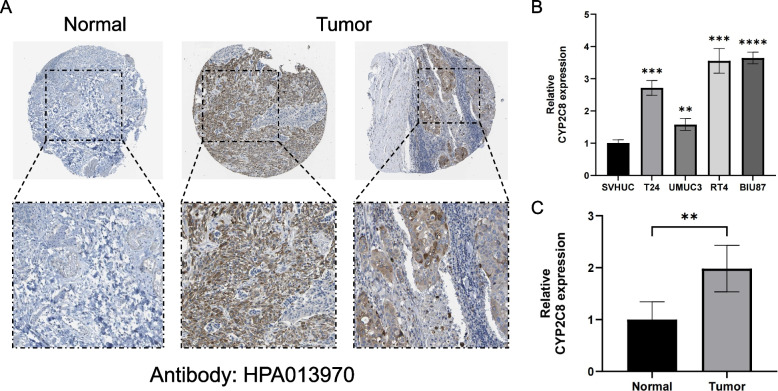
Expression validation of CYP2C8 (**A**) The immunohistochemistry image depicting the expression of CYP2C8 in normal bladder tissue and BLCA tissue (**B**) Differences in mRNA expression of CYP2C8 in normal and BLCA cell lines (**C**) Differences in mRNA expression of CYP2C8 in normal and BLCA tissues

In the previous differential gene expression analysis, CYP2C8 was extracted as a gene whose expression was upregulated in bladder cancer. Utilizing immunohistochemistry images from the HPA database, our analysis focused on comparing BLCA tissue to normal bladder tissue to confirm the result at the protein level (Fig. 11 A). Furthermore, encouragingly, qRT-PCR of cell lines as well as tissue samples verified higher expression of CYP2C8 in bladder cancer (Fig. 11 B-C). These analyses consistently affirmed the enhanced expression of CYP2C8 in bladder cancer samples, further emphasizing its potential importance in this disease.

### Core gene-based pan-cancer analysis

**Fig. 12:**
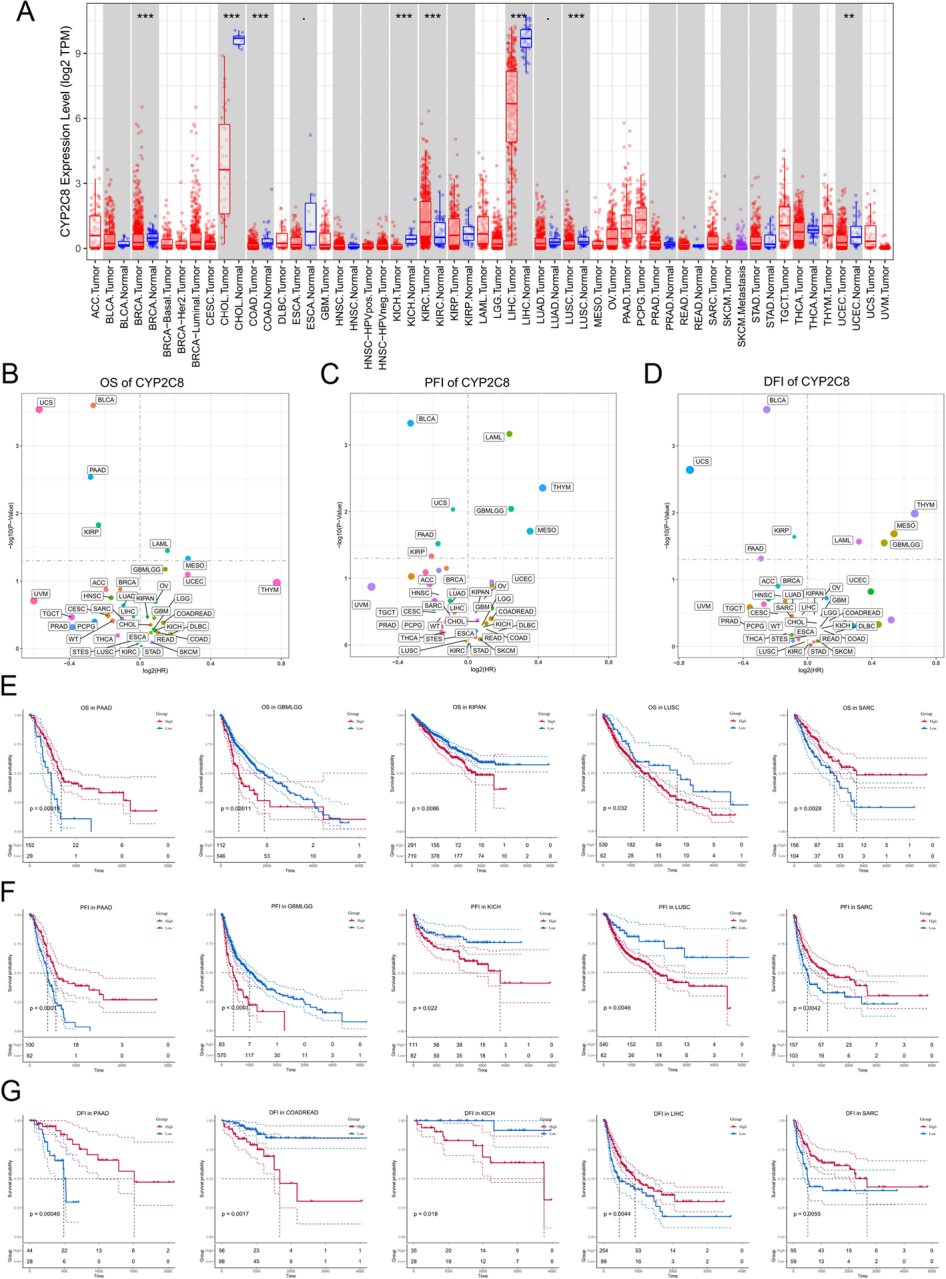
Prognostic potential of CYP2C8 in pan-cancer. **A** Differential pan-cancer expression of CYP2C8 (**B-D**) Prognostic potential of CYP2C8 for OS (**B**), PFI (**C**) and DFI (**D**). **E–G** KM curves of CYP2C8 differentiating OS (**E**), DFI (**F**) and PFI (**G**) in selected cancer types

**Fig. 13:**
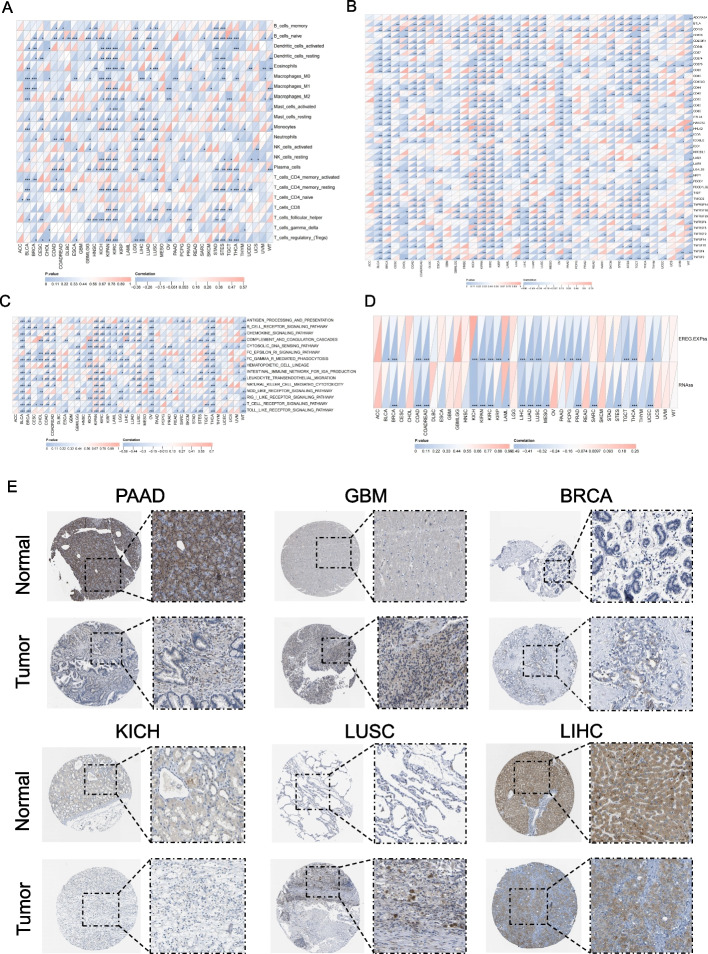
Pan-cancer functional analysis and expression validation of CYP2C8 (**A-D**) Correlation of CYP2C8 expression with pan-cancer immune cell infiltration (**A**), immune checkpoint expression (**B**), immunoinflammation-related functions (**C**) and stemness score (**D**).** E** Immunohistochemical picture of CYP2C8 in some cancer types

We searched a large number of literatures and found that few studies have investigated CYP2C8 as a tumor biomarker, so we attempted to perform a pan-cancer analysis of it. Initially, we showed the pan-cancer expression landscape of CYP2C8 (Fig. 12 A). Then by univariate cox analysis, CYP2C8 was determined to be significantly associated with OS, DFI and PFI in a range of cancers, such as PAAD, KIRP, GBMLGG, etc. (Fig. 12 B-D). Subsequently, KM curves were utilized to more visually demonstrate the prognostic potential of CYP2C8 in a number of cancer types with significant survival differences between high and low expression groups (Fig. 12 E–G). In addition, we shed some light on the potential regulatory direction of CYP2C8. We accordingly analyzed the correlation of CYP2C8 expression with immune cell infiltration (Fig. 13 A), immune checkpoint expression (Fig. 13 B), immune inflammation-related functions (Fig. 13 C) and stemness score (Fig. 13 D). We have a number of important findings, such as the fact that almost all immune checkpoint genes in KIRC showed a positive correlation with CYP2C8, whereas the opposite was true in KICH. Finally, we comparatively verified the differential expression of CYP2C8 in some cancer types by immunohistochemical profiles derived from the HPA database (Fig. 13 E).

## Discussion

BLCA, a prevalent and highly aggressive malignancy, significantly undermines patients' overall well-being [43]. It is of paramount importance to develop effective prognostic tools that can guide treatment decisions and optimize patient outcomes in BLCA. Recent endeavors have introduced numerous prognostic frameworks for BLCA, encompassing those reliant on genes tied to propionate metabolism [31], glycolysis [32], as well as ferroptosis- [33] and dendritic cell-related genetic signatures [44]. These frameworks have deepened our understanding of the prognostic trajectory of BLCA, laying a solid foundation for clinical interventions. Although ADME genes are recognized for their prognostic relevance in renal cancer [24, 25], non-small-cell lung cancer [28] and head and neck squamous cell carcinoma [27], their prognostic utility in BLCA is yet to be confirmed, showing restricted applicability in this context. Our investigation introduces a novel paradigm that sheds new light on prognostic evaluation and clinical management approaches for BLCA patients.

Bioinformatics, as a sharp edge of scientific research in the information age, provides many facilities and techniques for researchers to explore in depth. In this regard, we are greatly inspired by the scientific content of Liu's lab. For example, discovering the role of single gene in a single tumor, they argued in detail the identity of SCN3B and CDK2 in glioma [36, 38], and proved the role of AIMP1 [40], CNIH4 [41] in head and neck squamous cell carcinoma. As for the regulation of gene sets in single cancers, Liu et al. collated the prognostic potential of CD8 T-cell markers in breast cancer [34], emphasizing the impact of the IGFBP family in gliomas [45]. In addition, single-gene pan-cancer studies have also inspired us, such as the discovery of the broad diagnostic and prognostic value of RAD51 [39], TRPM7 [46], and CENPA [47] in pan-cancer. Finally, the pan-cancer analysis of gene sets is more prospective, which will be the focus of our future research. In this regard, voltage-gated sodium channels [48] and disulfidptosis-related genes [49], have a wide range of regulatory effects in pan-cancer, which are worth exploring in depth.

Our analysis revealed that over a quarter (80/298, 26.8%) of ADME genes display aberrant expression patterns in tumor tissues, hinting at their crucial role in cancer initiation and progression. Employing univariate Cox regression and lasso regression methodologies [45], we pinpointed 17 ADME genes with prognostic implications: CYP2C8, SLC22A1, ABCA4, ABCC9, CHST6, CYP1B1, CYP27B1, CYP3A43, CYP3A7, CYP4F8, NR1I2, PON1, SLC22A11, SLC22A3, SULT1A3, TAP1, and UGT2B4. Current research underscores the involvement of ADME genes in tumorigenesis. Notably, CYP2C8, a pivotal element in our constructed PPI network, was conspicuously upregulated in BLCA tissues. Qu et al. notes a pronounced correlation between CYP2C8 polymorphisms and heightened bladder cancer vulnerability [50]. Moreover, CYP2C8, a key gene for iron metabolism, is closely related to the prognosis as well as the treatment of BLCA patients [51]. Additionally, transport proteins like SLC22A1, SLC22A3, and SLC22A11, belonging to the organic cation transporter (OCT) family, play a critical role in modulating the toxicity and effectiveness of platinum-based chemotherapy treatments [52]. ABC transporter superfamily members, ABCA4 and ABCC9, showing the highest mutation rate in the 17 prognostic genes, have been implicated in breast cancer prognosis and drug response [53, 54]. CHST6, a carbohydrate sulfotransferase-encoding gene, is associated with macular corneal dystrophy [55, 56] and may function as a prognostic indicator for low-grade gliomas [57]. NR1I2, a xenobiotic-sensing nuclear receptor, halts liver cancer progression by inhibiting EMT in hepatocellular carcinoma [58]. PON1 has emerged as a prognostic biomarker for hepatocellular carcinoma [59]. TAP1, critical for MHC class I antigen processing and display, exhibits elevated expression across multiple cancer types and holds significant prognostic value [60]. Variations in the UGT2B4 gene are linked to an increased likelihood of developing pancreatic cancer and influence the pathological outcomes of chemotherapy in patients with breast cancer [61, 62]. CYP1B1 promotes metastatic cancer cell growth in a fatty acid-dependent manner [63]. CYP27B1, vital for steroid biosynthesis, curbs ovarian cancer cell proliferation, migration, and invasion [64]. The CYP3A family contributes to the transition from chronic atrophic gastritis to gastric cancer and modulates chemotherapeutic metabolism and resistance [65]. Lastly, dopamine-mediated upregulation of SULT1A3 markedly enhances the metastatic potential of hepatocellular carcinoma cells [66].

We harnessed these 17 ADME genes associated with prognosis to devise a risk assessment metric for each specimen and constructed a predictive framework. In both the training and validation groups, individuals identified as high-risk and low-risk exhibited markedly different survival paths. Demonstrated by ROC curves, the efficacy of our model to predict overall survival was convincingly established. In addition, the model's accurate predictive effect was verified again in cohorts with different clinical characteristics. Furthermore, both univariable and multivariable Cox regression analyses independently confirmed the reliability of our risk metric as a predictor of outcomes in bladder cancer. Additionally, we crafted a nomogram that seamlessly integrates the ADME-based risk metric with clinical indices. And calibration, DCA and ROC curve analyses substantiate the exemplary prognostic performance showcased.

In addition, the choice of tools for assessing the prognostic ability of the model deserves an in-depth discussion. The Kaplan–Meier (KM) survival curve is a widely used nonparametric method for survival analysis, which can visualize differences in survival among different groups, but has some limitations. The first thing to note is that the KM curve is only suitable for univariate group comparisons, so when we need to include covariates in the analysis, Cox regression models can be utilized, which is also reflected in our study. Secondly, when the sample size included in the analysis is small, the KM curves are assessed to be unstable as the confidence intervals become wider. This situation can be considered by using Bayesian methods to introduce prior information to enhance the estimation stability. In addition, the KM curve analysis assumes that censoring is not related to event risk, but in reality, there may be informative censoring due to deterioration of the condition, which may result in biased results. We can try to perform a sensitivity analysis to assess the effect of the censoring mechanism on the results, or construct a competing risk model for assessment when there is competition for events (e.g., death or relapse). In addition to the KM Survival Curve, both the Receiver Operating Characteristic (ROC) Curve and Decision Curve Analysis (DCA) were implemented in this study to assess model capability, with different and complementary focuses.The ROC Curve complements each other by Area Under Curve ( AUC) to quantify the discriminatory power of the model, which is very intuitive and can also be combined with survival time to reflect the predictive power of the model at different time points. However, the AUC only reflects the statistical performance of the model and fails to take into account the actual needs of clinical decision-making, which is complemented by the DCA, which evaluates the clinical utility of the model by calculating the net benefit, and determines whether the model's use is superior to the default strategy of all-or-nothing intervention. At the same time, however, DCA ignores the accuracy of the model, so calibration curves or ROC curves are often co-opted to verify that the predicted probabilities are consistent with the actual risks. Co-using ROC curves and DCA realizes the unity of science and practicality and advances the clinical translation of the model.

Our study examined the functional annotations of genes that were expressed differently between risk groups, aiming to elucidate the role of ADME genes in BLCA. Gene Ontology enrichment analyses illuminated an overrepresentation of functions tied to the extracellular matrix (ECM) and signaling cascades, embracing ECM organization, extracellular structural architecture, collagen-enriched ECM frameworks, signaling receptor activation, and receptor-ligand interactions. The tumor microenvironment (TME) relies fundamentally on ECM, which is made up of a complex variety of proteins secreted by cells. These proteins are essential for maintaining structural integrity and enabling detailed cellular interactions [67]. Perturbations in ECM levels can directly alter its biological attributes, thereby fostering cancer dissemination via tumor cell heterogeneity [68]. KEGG demonstrates enrichment for many notorious pro-oncogenic pathways, such as PI3K-AKT signaling pathway and focal adhesion. Over-regulation of the PI3K-AKT pathway tends to mediate malignant cell proliferation and promotes the manifestation of cancer phenotypes [69]. In addition to mediating cell adhesion and promoting cancer cell proliferation, adhesion plaques also have significant effects on stemness regulation and cell proliferation [70]. GSEA, on the other hand, highlights the importance of immune-related pathways between high and low risk groups.

Our exhaustive investigation unveiled disparities in immune cell infiltration patterns across risk groups. Our observations highlighted a notable surge in M2 macrophages within the high-risk cohort, with the risk metric directly mirroring the abundance of all macrophage subtypes. Converging evidence underscores the association between elevated tumor-associated macrophages (TAMs) and unfavorable survival outcomes, as well as compromised therapeutic responses to chemotherapy and BCG therapy [71–73]. Notably, M2 macrophages are implicated in dampening immune responses and fostering angiogenesis in bladder cancer, thereby expediting tumor progression and dissemination [74]. This analysis indicates that the presence of M2 macrophages correlates with poorer outcomes among individuals in the high-risk category.In the group identified as high-risk, a pronounced deficit was observed in both activated dendritic cells and natural killer cells. Notably, the dendritic cell count inversely correlated with the risk metrics, underscoring a substantial imbalance in immune cell distribution. Given the pivotal role of dendritic cells in activating NKT and γδT cells to combat bladder cancer cells [75], the diminished occurrence of these cells may be a factor in the poorer outcomes observed in the high-risk cohort. Contrary to this, elevated levels of CD8 T cells in the low-risk group align with previous findings [76], suggesting that greater infiltration of these cells improves patient outcomes. And when we focused on the regulation of immune infiltration by individual model genes, it has been reported that the peroxidase-like activity of PON1 inhibits the differentiation of monocytes to macrophages [77]. In our model, PON1 expression was higher in the low-risk group, and cibersort analysis showed less macrophage infiltration in the low-risk group, and PON1 expression was negatively correlated with M0 macrophages, in agreement with the above literature. Moreover, TAP1 expression promotes cytotoxic T cell infiltration [78], whereas TAP1-deficient mice exhibit reduced expression of HLA class I molecules and thus a severe lack of CD8 T cells [79]. This is consistent with the higher expression of TAP1 as well as higher abundance of CD8 T cells in the low-risk group in our model. In addition to the above, there are fewer studies on the mechanism of model genes and immune cell infiltration. Although our study revealed a strong association between ADME genes and immune cell infiltration, the specific regulatory mechanisms remain unclear. Regulation of individual model genes Given the complexity of the immune microenvironment, the effect of individual genes may not fully explain the extent of immune cell infiltration, so in the future, we will try to conduct more precise controlled variable experiments and obtain samples from high- and low-risk patients from the clinic for detailed mechanism analysis.

Exploring correlations uncovered a pronounced inverse association between the risk metric and several immune checkpoint genes, such as CD40, TNFRSF14, TNFRSF25. CD40, a familiar immune checkpoint, is abundantly expressed on antigen-presenting cells, macrophages, and even tumor cells, where it interacts with helper T cells and mediates the enhancement of anti-tumor T cell responses [80]. In addition, it has been reported that CD40 stimulation of macrophages directly mediates cancer cell killing as well as microenvironment collapse [81]. The negative correlation index of TNFRSF14 and TNFRSF25 with risk scores was as low as −0.4. TNFRSF14 was associated with a better prognosis through promotion of apoptosis in BLCA [82], whereas TNFRSF25 mediated toxic killing of CD8 T cells to exert antitumor immunity [83]. Combined with our findings of upregulation of classical HLA expression and functional upregulation of MHC class I in patients in the low-risk group, the feasibility of diversified immunotherapies is highlighted. Our subsequent research illuminated a more favorable response to immunotherapy among low-risk patients within the IMvigor210 cohort. Our study revealed marked disparities in the tumor immune microenvironment among different risk categories, highlighting its importance for prognostic evaluations and the clinical management of bladder cancer. While focusing on specific model component genes, we found that fewer studies on the exact mechanism were reported. Among them, TAP1 was identified as a reliable prognostic target for pan-cancer immunotherapeutic response [60], which increases PDL1 expression and can be enhanced by oxaliplatin stimulation [84]. In addition, in colorectal cancer, Chen et al. found that CYP1B1 could trigger anti-PD-1 immunotherapy inactivation by mediating ubiquitination degradation of ACSL4 [85]. In conclusion many ADME genes may play a potentially influential role in cancer immunotherapy. Future endeavors should delve deeper into the intricate interplay among ADME genes, immune cell dynamics, and the immunotherapeutic response, with the aim of enhancing patient outcomes.

Malignant phenotypic correlation analysis showed that our established risk score showed a highly positive correlation with angiogenesis as well as EMT, which contributes to an in-depth understanding of the root causes of the progression of poorer tumor prognosis. Interestingly, we found that TMB showed a significant negative correlation with the risk score, implying that the low-risk group had a higher propensity for mutation. Previous study [86] have elaborated that antigenic changes due to mutations promote immune cell recognition and presentation, which mediates cell killing, resulting in greater benefit from immunotherapy. This is another aspect that confirms the better prognosis of immunotherapy in low-risk patients that we found in the above study.

To refine clinical outcomes, we performed a drug sensitivity assessment, revealing distinct response patterns between risk groups. Our research reveals that patients with high-risk profiles generally exhibit positive responses to traditional medications such as Foretinib and Dasatinib. Conversely, these same groups show resistance to drugs like Oxaliplatin, Gefitinib, and Cyclophosphamide. When we looked at the mechanism by which specific ADME model genes affect chemotherapy response, it had been reported that overexpression of CYP1B1 mediates complex cancer cell phenotypes such as enhanced mesenchymalization, which led to resistance to chemotherapeutic agents such as 5-fluorouracil, cisplatin, and others [87]. In addition, platinum-based chemotherapeutic agents such as oxaliplatin had been reported to be substrates for SLC22A1-3 [88], and high expression of SLC22A3 promoted the uptake of oxaliplatin, which accumulated more platinum and exerted stronger toxic effects on cancer cells [89]. However, in our study, the trend of differential expression of SLC22A1-3 in the high- and low-risk groups varied, so the specific regulatory mechanism threw further control variables need to be further controlled variables in-depth study. Moreover, the use of chemotherapeutic agents also had an impact on the activity of the ADME gene protein, such as Gefetinib and other kinase inhibitors, which were inhibitors of the activity of CYP3A [90]. We speculated that the reduction of the activity of the ADME protein may also subsequently regulate the efficacy of the chemotherapeutic agents in a reverse manner. There are few reports on the regulatory mechanisms of specific ADME genes in chemotherapy, so future research should focus on this aspect to further advance customized treatment strategies and combination chemotherapy regimens.

CYP2C8 was extracted as a core target by us, and by qRT-PCR with immunohistochemistry, we perfectly validated the differential expression conclusion obtained from bioinformatics analysis. In our analysis, high expression of CYP2C8 predicted a better prognosis for BLCA patients, suggesting that CYP2C8 may mediate some protective mechanisms. Notably, high expression of CYP2C8 was associated with higher infiltration of dendritic cells, which, as the mainstay of antigen presentation, have been reported to positively contribute to tumor immunity in bladder cancer [76]. We speculate that CYP2C8 may contribute in regulating dendritic cells, mediating a better prognosis by modulating the immune microenvironment. GSVA heatmap showed that the high expression group exhibited higher peroxisome activity. One of the major functions of peroxisomes is to metabolize reactive oxygen species [91], which act as active second messengers in the cell and are involved in proliferation, angiogenesis, and other notorious pro-carcinogenic processes [92]. We speculate that CYP2C8 may mediate peroxisome-associated functions to stabilize the intracellular reactive oxygen species environment, thereby reducing the malignancy of cancer cells to a certain extent and leading to a better prognosis. In addition, there were significant metabolic pathway differences between the high and low expression groups of CYP2C8, which may also have an impact on cancer development. We then saw the light and pushed CYP2C8 from bladder cancer to pan-cancer and revealed its potential as a novel prognostic target through comprehensive immune-related and stemness-related analyses. Although CYP2C8 is closely associated with the prognosis of bladder cancer and even pan-cancer, the specific regulatory mechanism remains unclear. In the future, we will focus on the identity of CYP2C8 in the metabolic microenvironment and immune microenrivronment of cancer cells and explore the specific mechanism by which it affects cancer progression.

Personalized medicine is an increasingly popular model of care for clinicians, aiming to provide the most rational healthcare plan by integrating the patient's multi-omic data, clinical characteristics, physiological conditions, living environment and even economic status [93]. Personalized medicine is particularly important in the management of BLCA, a urologic tumor with significant risk heterogeneity, where cancer progression, genomic landscapes, and immune microenvironments vary from patient to patient [94, 95]. With an increasing number of drug candidates for BLCA [96], the selection of rational therapies is a pressing issue [97], and by utilizing the differences in drug sensitivities obtained from the risk stratification of our model, clinicians can attempt to formulate precise and targeted therapeutic strategies. For patients identified as high-risk, they may show higher sensitivity to Staurosporine, Dasatinib, and thus these drugs can be selected as first-line treatment options. In addition, for the treatment of patients in the high-risk group, it is necessary for clinicians to work together in a joint multidisciplinary team (MDT) because the comprehensive management of patients with BLCA, including physical, nutritional, and emotional aspects, is considered to be a crucial part of the treatment process [98]. On the other hand, high-risk patients may show a tendency to have a worse prognosis, which highlights the importance of choosing the desired outcome [99] and may require the cooperation of the medical staff for appropriate end-of-life care. For patients identified as low risk, according to our model, may show higher sensitivity to anti-PDL1 immunotherapy as well as Oxaliplatin, 5-Fluorouracil chemotherapy. Risk modeling should be used for ongoing prognostic assessment while clinicians administer targeted therapies based on protocols. When periodic pathology and tumor marker assessments determine that the tumor is likely to progress, clinicians can use risk modeling to rapidly reassess the patient's changing risk of regression and adjust treatment strategies accordingly. With our risk model, clinicians have a reference point to implement more precise treatments rather than relying on empirical or non-specific approaches, thus facilitating the process of personalized management of BLCA and laying a solid foundation for ensuring a better prognosis for patients.

While our findings are encouraging, several limitations must be acknowledged. The reliance on publicly available datasets (mainly TCGA and GEO) may introduce biases related to sample selection and population characterization, which will lead to a lack of model generalizability within large groups. For example, the TCGA database used for model training focuses on European and American populations and lacks ethnic diversity such as Asian and African populations, leading to biases in genetic background, environmental exposure, and other factors. Moreover, the samples are mainly early-stage cancer patients, with relatively few advanced and metastatic cases, which limits the applicability of the results to advanced cancers to a certain extent. And the GEO dataset is mainly expression profiles from high-throughput sequencing, which is not a comprehensive record of patients' clinical characteristics, drug treatment response, immunotherapy effect and other parameters, resulting in a lack of clinical efficacy exploration of the model. Therefore, we are trying to construct a more comprehensive BLCA cohort, including the inclusion of patients' treatment information, to address these limitations, further explore the generalizability of the model, and lay the foundation for more detailed mechanistic analysis. In addition, technical and biological biases in bioinformatics analyses are important factors contributing to the limitations [100, 101]. Limitations in technical factors are mainly caused by sequencing methods, such as for microarray chips microarray chips, where similar sequences may hybridize with the wrong probes, leading to false signal detection or signal interference. And RNA sequencing may have problems such as PCR amplification bias and differences in sequencing depth and coverage. To cope with these limitations, we can take a specific approach to specific data, such as analyzing microarray data with the “limma” package, and analyzing RNA sequencing data with “DESeq2” or “edgeR” packages. RNA sequencing data are analyzed with “DESeq2” or “edgeR”, and the sequencing depth, batch effect and amplification bias are adjusted by normalization procedures. It is also worth noting that cross-platform batch effects can mask the true biological signal, so when combining datasets from multiple platforms for analysis, the “sva” package can be used for de-batching. Limitations due to biological factors are mainly due to intra-tumor heterogeneity, sample purity, etc. Bulk RNA-seq averages the signals from all cells in the sample and is not able to show the intra-tumor expression landscape at a fine resolution. Therefore, the detailed molecular landscape can be further explored in the future by means of single-cell sequencing and other means. Finally, the validation of the experiments is a very direct guide to the bioinformatics findings, and more in-depth mechanistic experiments should be conducted in the future to explore the role of ADME genes in the progression of BLCA.

## Conclusion

Our research established and confirmed a prognostic risk assessment tool based on ADME gene utilization, which showed high predictive accuracy for survival rates in bladder cancer. Functional enrichment analyses revealed unique immune-related signatures across high- and low-risk patient groups, connecting ADME gene expression patterns with the complex dynamics of the tumor microenvironment. Furthermore, the integration of clinical factors into a nomogram serves as a valuable instrument for precise prognosis prediction, enabling tailored treatment strategies for BLCA patients, thereby advancing personalized medicine. In addition, we identified CYP2C8 as a novel BLCA target and generalized it to pan-cancer.

## Supplementary Information

Supplementary Material 1.

Supplementary Material 2.

Supplementary Material 3.

## Data Availability

No datasets were generated or analysed during the current study.

## References

[1] Sung H, Ferlay J, Siegel RL, Laversanne M, Soerjomataram I, Jemal A, Bray F. Global Cancer Statistics 2020: GLOBOCAN Estimates of Incidence and Mortality Worldwide for 36 Cancers in 185 Countries. CA Cancer J Clin. 2021;71(3):209–49. 10.3322/caac.21660. 33538338 10.3322/caac.21660

[2] Al-Zalabani AH, Stewart KF, Wesselius A, Schols AM, Zeegers MP. Modifiable risk factors for the prevention of bladder cancer: a systematic review of meta-analyses. Eur J Epidemiol. 2016;31(9):811–51. 10.1007/s10654-016-0138-6. 27000312 10.1007/s10654-016-0138-6 PMC5010611

[3] Phelan A, Lopez-Beltran A, Montironi R, Zhang S, Raspollini MR, Cheng M, Kaimakliotis HZ, Koch MO, Cheng L. Inherited forms of bladder cancer: a review of Lynch syndrome and other inherited conditions. Future Oncol. 2018;14(3):277–90. 10.2217/fon-2017-0346. 29345160 10.2217/fon-2017-0346

[4] van Osch FH, Jochems SH, van Schooten FJ, Bryan RT, Zeegers MP. Quantified relations between exposure to tobacco smoking and bladder cancer risk: a meta-analysis of 89 observational studies. Int J Epidemiol. 2016;45(3):857–70. 10.1093/ije/dyw044. 27097748 10.1093/ije/dyw044

[5] Cumberbatch MG, Cox A, Teare D, Catto JW. Contemporary Occupational Carcinogen Exposure and Bladder Cancer: A Systematic Review and Meta-analysis. JAMA Oncol. 2015;1(9):1282–90. 10.1001/jamaoncol.2015.3209. Erratum in: JAMA Oncol. 2015 Dec;1(9):1224. 10.1001/jamaoncol.2015.5120. 26448641 10.1001/jamaoncol.2015.3209

[6] Siegel RL, Giaquinto AN, Jemal A. Cancer statistics, 2024. CA Cancer J Clin. 2024;74(1):12–49. 10.3322/caac.21820. 38230766 10.3322/caac.21820

[7] Stecca C, Abdeljalil O, Sridhar SS. Metastatic Urothelial Cancer: a rapidly changing treatment landscape. Ther Adv Med Oncol. 2021;13:17588359211047352. 10.1177/17588359211047352. 34616491 10.1177/17588359211047352 PMC8488509

[8] Tambaro R, Napoli MD, Pisano C, Cecere SC, Attademo L, Rossetti S, Feroce F, Setola S, Califano D, Russo D, Spina A, Perdonà S, Izzo A, Pignata S. From clinical trials to clinical use of checkpoint inhibitors for patients with metastatic urothelial cancer. Immunotherapy. 2021;13(1):67–77. 10.2217/imt-2020-0128. 33045887 10.2217/imt-2020-0128

[9] Sonkin D, Thomas A, Teicher BA. Cancer treatments: Past, present, and future. Cancer Genet. 2024;286–287:18–24. 10.1016/j.cancergen.2024.06.002. 38909530 10.1016/j.cancergen.2024.06.002 PMC11338712

[10] Tao L, Zhang P, Qin C, Chen SY, Zhang C, Chen Z, Zhu F, Yang SY, Wei YQ, Chen YZ. Recent progresses in the exploration of machine learning methods as in-silico ADME prediction tools. Adv Drug Deliv Rev. 2015;86:83–100. 10.1016/j.addr.2015.03.014. 26037068 10.1016/j.addr.2015.03.014

[11] Gleeson MP, Hersey A, Montanari D, Overington J. Probing the links between in vitro potency, ADMET and physicochemical parameters. Nat Rev Drug Discov. 2011;10(3):197–208. 10.1038/nrd3367. 21358739 10.1038/nrd3367 PMC6317702

[12] Klein K, Tremmel R, Winter S, Fehr S, Battke F, Scheurenbrand T, Schaeffeler E, Biskup S, Schwab M, Zanger UM. A New Panel-Based Next-Generation Sequencing Method for ADME Genes Reveals Novel Associations of Common and Rare Variants With Expression in a Human Liver Cohort. Front Genet. 2019;10:7. 10.3389/fgene.2019.00007. 30766545 10.3389/fgene.2019.00007 PMC6365429

[13] Hu DG, Marri S, McKinnon RA, Mackenzie PI, Meech R. Deregulation of the Genes that Are Involved in Drug Absorption, Distribution, Metabolism, and Excretion in Hepatocellular Carcinoma. J Pharmacol Exp Ther. 2019;368(3):363–81. 10.1124/jpet.118.255018. 30578287 10.1124/jpet.118.255018

[14] Hovelson DH, Xue Z, Zawistowski M, Ehm MG, Harris EC, Stocker SL, Gross AS, Jang IJ, Ieiri I, Lee JE, Cardon LR, Chissoe SL, Abecasis G, Nelson MR. Characterization of ADME gene variation in 21 populations by exome sequencing. Pharmacogenet Genomics. 2017;27(3):89–100. 10.1097/FPC.0000000000000260. 27984508 10.1097/FPC.0000000000000260 PMC5287433

[15] Jittikoon J, Mahasirimongkol S, Charoenyingwattana A, Chaikledkaew U, Tragulpiankit P, Mangmool S, Inunchot W, Somboonyosdes C, Wichukchinda N, Sawanpanyalert P, He Y, McLeod HL, Chantratita W. Comparison of genetic variation in drug ADME-related genes in Thais with Caucasian, African and Asian HapMap populations. J Hum Genet. 2016;61(2):119–27. 10.1038/jhg.2015.115. 26423926 10.1038/jhg.2015.115

[16] Arbitrio M, Di Martino MT, Scionti F, Barbieri V, Pensabene L, Tagliaferri P. Pharmacogenomic Profiling of ADME Gene Variants: Current Challenges and Validation Perspectives. High Throughput. 2018;7(4):40. 10.3390/ht7040040. 30567415 10.3390/ht7040040 PMC6306724

[17] Figueroa JD, Malats N, García-Closas M, et al. Bladder cancer risk and genetic variation in AKR1C3 and other metabolizing genes. Carcinogenesis. 2008;29(10):1955–62. 10.1093/carcin/bgn163. 18632753 10.1093/carcin/bgn163 PMC2556968

[18] Liu CY, Hsu YH, Pan PC, Wu MT, Ho CK, Su L, Xu X, Li Y, Christiani DC, Kaohsiung Leukemia Research Group. Maternal and offspring genetic variants of AKR1C3 and the risk of childhood leukemia. Carcinogenesis. 2008;29(5):984–90. 10.1093/carcin/bgn071. 18339682 10.1093/carcin/bgn071 PMC2902386

[19] Rehman K, Iqbal Z, Zhiqin D, Ayub H, Saba N, Khan MA, Yujie L, Duan L. Analysis of genetic biomarkers, polymorphisms in ADME-related genes and their impact on pharmacotherapy for prostate cancer. Cancer Cell Int. 2023;23(1):247. 10.1186/s12935-023-03084-5. 37858151 10.1186/s12935-023-03084-5 PMC10585889

[20] Tęcza K, Kalinowska-Herok M, Rusinek D, Zajkowicz A, Pfeifer A, Oczko-Wojciechowska M, Pamuła-Piłat J. Are the Common Genetic 3’UTR Variants in ADME Genes Playing a Role in Tolerance of Breast Cancer Chemotherapy? Int J Mol Sci. 2024;25(22):12283. 10.3390/ijms252212283. 39596349 10.3390/ijms252212283 PMC11594993

[21] Drozdzik M, Oswald S. Expression and Regulation of Drug Transporters and Metabolizing Enzymes in the Human Gastrointestinal Tract. Curr Med Chem. 2016;23(39):4468–89. 10.2174/0929867323666161024154457. 27781942 10.2174/0929867323666161024154457

[22] Fisel P, Schaeffeler E, Schwab M. DNA Methylation of ADME Genes. Clin Pharmacol Ther. 2016;99(5):512–27. 10.1002/cpt.343. 27061006 10.1002/cpt.343

[23] Wu KC, Lin CJ. The regulation of drug-metabolizing enzymes and membrane transporters by inflammation: Evidences in inflammatory diseases and age-related disorders. J Food Drug Anal. 2019;27(1):48–59. 10.1016/j.jfda.2018.11.005. 30648594 10.1016/j.jfda.2018.11.005 PMC9298621

[24] Wang H, Li F, Wang Q, Guo X, Chen X, Zou X, Yuan J. Identifying ADME-related gene signature for immune landscape and prognosis in KIRC by single-cell and spatial transcriptome analysis. Sci Rep. 2025;15(1):1294. 10.1038/s41598-024-84018-7. 39779746 10.1038/s41598-024-84018-7 PMC11711672

[25] Zhang H, Huang W, Chen M, Liu Y, Yan B, Mou S, Jiang W, Mei H. Research on molecular characteristics of ADME-related genes in kidney renal clear cell carcinoma. Sci Rep. 2024;14(1):16834. 10.1038/s41598-024-67516-6. 39039118 10.1038/s41598-024-67516-6 PMC11263354

[26] Hu DG, Mackenzie PI, Nair PC, McKinnon RA, Meech R. The Expression Profiles of ADME Genes in Human Cancers and Their Associations with Clinical Outcomes. Cancers (Basel). 2020;12(11):3369. 10.3390/cancers12113369. 33202946 10.3390/cancers12113369 PMC7697355

[27] Han K, Wang J, Qian K, Zhao T, Zhang Y. Establishment of non-small-cell lung cancer risk prediction model based on prognosis-associated ADME genes. Biosci Rep. 2021;41(10):BSR20211433. 10.1042/BSR20211433. 34522968 10.1042/BSR20211433 PMC8527211

[28] Tang X, Li R, Wu D, Wang Y, Zhao F, Lv R, Wen X. Development and Validation of an ADME-Related Gene Signature for Survival, Treatment Outcome and Immune Cell Infiltration in Head and Neck Squamous Cell Carcinoma. Front Immunol. 2022;13: 905635. 10.3389/fimmu.2022.905635. 35874705 10.3389/fimmu.2022.905635 PMC9304892

[29] Goldman MJ, Craft B, Hastie M, Repečka K, McDade F, Kamath A, Banerjee A, Luo Y, Rogers D, Brooks AN, Zhu J, Haussler D. Visualizing and interpreting cancer genomics data via the Xena platform. Nat Biotechnol. 2020;38(6):675–8. 10.1038/s41587-020-0546-8. 32444850 10.1038/s41587-020-0546-8 PMC7386072

[30] Kim WJ, Kim EJ, Kim SK, Kim YJ, Ha YS, Jeong P, Kim MJ, Yun SJ, Lee KM, Moon SK, Lee SC, Cha EJ, Bae SC. Predictive value of progression-related gene classifier in primary non-muscle invasive bladder cancer. Mol Cancer. 2010;9:3. 10.1186/1476-4598-9-3. 20059769 10.1186/1476-4598-9-3 PMC2821358

[31] Zheng F, Wang Z, Li S, Xiong S, Yuan Y, Zeng J, Tan Y, Liu X, Xu S, Fu B. Development of a propionate metabolism-related gene-based molecular subtypes and scoring system for predicting prognosis in bladder cancer. Eur J Med Res. 2024;29(1):393. 10.1186/s40001-024-01982-6. 39075554 10.1186/s40001-024-01982-6 PMC11285334

[32] Shen C, Suo Y, Guo J, Su W, Zhang Z, Yang S, Wu Z, Fan Z, Zhou X, Hu H. Development and validation of a glycolysis-associated gene signature for predicting the prognosis, immune landscape, and drug sensitivity in bladder cancer. Front Immunol. 2025;15:1430583. 10.3389/fimmu.2024.1430583. 39867879 10.3389/fimmu.2024.1430583 PMC11757262

[33] Ma J, Hu J, Zhao L, Wu Z, Li R, Deng W. Identification of clinical prognostic factors and analysis of ferroptosis-related gene signatures in the bladder cancer immune microenvironment. BMC Urol. 2024;24(1):6. 10.1186/s12894-023-01354-y. 38172792 10.1186/s12894-023-01354-y PMC10765654

[34] Liu H, Dong A, Rasteh AM, Wang P, Weng J. Identification of the novel exhausted T cell CD8 + markers in breast cancer. Sci Rep. 2024;14(1):19142. 10.1038/s41598-024-70184-1. 39160211 10.1038/s41598-024-70184-1 PMC11333736

[35] Tibshirani R. The lasso method for variable selection in the Cox model. Stat Med. 1997;16(4):385–95. 9044528 10.1002/(sici)1097-0258(19970228)16:4<385::aid-sim380>3.0.co;2-3

[36] Liu H, Weng J, Huang CL, Jackson AP. Is the voltage-gated sodium channel β3 subunit (SCN3B) a biomarker for glioma? Funct Integr Genomics. 2024;24(5):162. 10.1007/s10142-024-01443-7. 39289188 10.1007/s10142-024-01443-7

[37] Liu H, Tang T. MAPK signaling pathway-based glioma subtypes, machine-learning risk model, and key hub proteins identification. Sci Rep. 2023;13(1):19055. 10.1038/s41598-023-45774-0. 37925483 10.1038/s41598-023-45774-0 PMC10625624

[38] Liu H, Weng J. A comprehensive bioinformatic analysis of cyclin-dependent kinase 2 (CDK2) in glioma. Gene. 2022;822: 146325. 10.1016/j.gene.2022.146325. 35183683 10.1016/j.gene.2022.146325

[39] Liu H, Weng J. A Pan-Cancer Bioinformatic Analysis of RAD51 Regarding the Values for Diagnosis, Prognosis, and Therapeutic Prediction. Front Oncol. 2022;12: 858756. 10.3389/fonc.2022.858756. 35359409 10.3389/fonc.2022.858756 PMC8960930

[40] Li Y, Liu H. Clinical powers of Aminoacyl tRNA Synthetase Complex Interacting Multifunctional Protein 1 (AIMP1) for head-neck squamous cell carcinoma. Cancer Biomark. 2022;34(3):359–74. 10.3233/CBM-210340. 35068446 10.3233/CBM-210340 PMC12364190

[41] Liu H, Li Y. Potential roles of Cornichon Family AMPA Receptor Auxiliary Protein 4 (CNIH4) in head and neck squamous cell carcinoma. Cancer Biomark. 2022;35(4):439–50. 10.3233/CBM-220143. 36404537 10.3233/CBM-220143 PMC12364253

[42] Newman AM, Liu CL, Green MR, Gentles AJ, Feng W, Xu Y, Hoang CD, Diehn M, Alizadeh AA. Robust enumeration of cell subsets from tissue expression profiles. Nat Methods. 2015;12(5):453–7. 10.1038/nmeth.3337. 25822800 10.1038/nmeth.3337 PMC4739640

[43] Patel VG, Oh WK, Galsky MD. Treatment of muscle-invasive and advanced bladder cancer in 2020. CA Cancer J Clin. 2020;70(5):404–23. 10.3322/caac.21631. 32767764 10.3322/caac.21631

[44] An B, Guo Z, Wang J, Zhang C, Zhang G, Yan L. Derivation and external validation of dendritic cell-related gene signatures for predicting prognosis and immunotherapy efficacy in bladder urothelial carcinoma. Front Immunol. 2022;13:1080947. 10.3389/fimmu.2022.1080947. 36578478 10.3389/fimmu.2022.1080947 PMC9790929

[45] Liu H, Tang T. A bioinformatic study of IGFBPs in glioma regarding their diagnostic, prognostic, and therapeutic prediction value. Am J Transl Res. 2023;15(3):2140–55. 37056850 PMC10086936

[46] Liu H, Dilger JP, Lin J. A pan-cancer-bioinformatic-based literature review of TRPM7 in cancers. Pharmacol Ther. 2022;240: 108302. 10.1016/j.pharmthera.2022.108302. 36332746 10.1016/j.pharmthera.2022.108302

[47] Liu H, Karsidag M, Chhatwal K, Wang P, Tang T. Single-cell and bulk RNA sequencing analysis reveals CENPA as a potential biomarker and therapeutic target in cancers. PLoS ONE. 2025;20(1): e0314745. 10.1371/journal.pone.0314745. 39820192 10.1371/journal.pone.0314745 PMC11737691

[48] Liu H, Weng J, Huang CL, Jackson AP. Voltage-gated sodium channels in cancers. Biomark Res. 2024;12(1):70. 10.1186/s40364-024-00620-x. 39060933 10.1186/s40364-024-00620-x PMC11282680

[49] Liu H, Tang T. Pan-cancer genetic analysis of disulfidptosis-related gene set. Cancer Genet. 2023;278–279:91–103. 10.1016/j.cancergen.2023.10.001. 37879141 10.1016/j.cancergen.2023.10.001

[50] Qu W, Zhang F, Cheng Y, Li J, Zhou J. The impact of genetic variants in the CYP2C8 gene on bladder cancer susceptibility. Front Endocrinol (Lausanne). 2022;13: 989030. 10.3389/fendo.2022.989030. 36246885 10.3389/fendo.2022.989030 PMC9554954

[51] Song X, Xin S, Zhang Y, Mao J, Duan C, Cui K, Chen L, Li F, Liu Z, Wang T, Liu J, Liu X, Song W. Identification and Quantification of Iron Metabolism Landscape on Therapy and Prognosis in Bladder Cancer. Front Cell Dev Biol. 2022;10: 810272. 10.3389/fcell.2022.810272. 35265613 10.3389/fcell.2022.810272 PMC8899848

[52] Zhang S, Lovejoy KS, Shima JE, Lagpacan LL, Shu Y, Lapuk A, Chen Y, Komori T, Gray JW, Chen X, Lippard SJ, Giacomini KM. Organic cation transporters are determinants of oxaliplatin cytotoxicity. Cancer Res. 2006;66(17):8847–57. 10.1158/0008-5472.CAN-06-0769. 16951202 10.1158/0008-5472.CAN-06-0769 PMC2775093

[53] Chen Q, Zhou Q. Identification of exosome-related gene signature as a promising diagnostic and therapeutic tool for breast cancer. Heliyon. 2024;10(8): e29551. 10.1016/j.heliyon.2024.e29551. 38665551 10.1016/j.heliyon.2024.e29551 PMC11043961

[54] Hlaváč V, Václavíková R, Brynychová V, Koževnikovová R, Kopečková K, Vrána D, Gatěk J, Souček P. Role of Genetic Variation in ABC Transporters in Breast Cancer Prognosis and Therapy Response. Int J Mol Sci. 2020;21(24):9556. 10.3390/ijms21249556. 33334016 10.3390/ijms21249556 PMC7765380

[55] Muthana SM, Campbell CT, Gildersleeve JC. Modifications of glycans: biological significance and therapeutic opportunities. ACS Chem Biol. 2012;7(1):31–43. 10.1021/cb2004466. 22195988 10.1021/cb2004466 PMC3262866

[56] Hao XD, Liu YN, Hu SH, Pan XJ, Chen P. Association of macular corneal dystrophy with excessive cell senescence and apoptosis induced by the novel mutant CHST6. Exp Eye Res. 2022;214: 108862. 10.1016/j.exer.2021.108862. 34826417 10.1016/j.exer.2021.108862

[57] Liu G, Lu Y, Gao D, Huang Z, Ma L. Identification of an energy metabolism-related six-gene signature for distinguishing and forecasting the prognosis of low-grade gliomas. Ann Transl Med. 2023;11(3):146. 10.21037/atm-22-6502. 36846014 10.21037/atm-22-6502 PMC9951020

[58] Sato T, Shizu R, Baba R, Ooka A, Hosaka T, Kanno Y, Yoshinari K. Pregnane X receptor inhibits the transdifferentiation of hepatic stellate cells by down-regulating periostin expression. Biochem J. 2024;481(18):1173–86. 10.1042/BCJ20240172. 39171361 10.1042/BCJ20240172

[59] Dong L, Dong C, Yu Y, Jiao X, Zhang X, Zhang X, Li Z. Transcriptomic analysis of Paraoxonase 1 expression in hepatocellular carcinoma and its potential impact on tumor immunity. Clin Transl Oncol. 2024. 10.1007/s12094-024-03598-y. 39031295 10.1007/s12094-024-03598-y

[60] Tu Z, Li K, Ji Q, Huang Y, Lv S, Li J, Wu L, Huang K, Zhu X. Pan-cancer analysis: predictive role of TAP1 in cancer prognosis and response to immunotherapy. BMC Cancer. 2023;23(1):133. 10.1186/s12885-022-10491-w. 36759763 10.1186/s12885-022-10491-w PMC9912572

[61] Che X, Yu D, Wu Z, Zhang J, Chen Y, Han Y, Wang C, Qi J. Polymorphisms in UGT2B4 and susceptibility to pancreatic cancer. Int J Clin Exp Med. 2015;8(2):2702–10. 25932223 PMC4402870

[62] Gao QL, Zhang XF, Geng L, Tian TD, Sun XF. Effect of single nucleotide polymorphisms of RS1826690 located in UGT2B4 gene on the pathological complete response to neoadjuvant chemotherapy in breast cancer patients. Zhonghua Yi Xue Za Zhi. 2018;98(16):1242–5. 10.3760/cma.j.issn.0376-2491.2018.16.011. Chinese. 29747312 10.3760/cma.j.issn.0376-2491.2018.16.011

[63] Jin L, Huang J, Guo L, Zhang B, Li Q, Li H, Yu M, Xie P, Yu Q, Chen Z, Liu S, Xu Y, Xiao Y, Lu M, Ye Q. CYP1B1 promotes colorectal cancer liver metastasis by enhancing the growth of metastatic cancer cells via a fatty acids-dependent manner. J Gastrointest Oncol. 2023;14(6):2448–65. 10.21037/jgo-23-895. 38196537 10.21037/jgo-23-895 PMC10772677

[64] Huo X, Sun H, Qian Q, Ma X, Peng P, Yu M, Zhang Y, Yang J, Cao D, Gui T, Shen K. CYP27B1 Downregulation: A New Molecular Mechanism Regulating EZH2 in Ovarian Cancer Tumorigenicity. Front Cell Dev Biol. 2020;8: 561804. 10.3389/fcell.2020.561804. 33163485 10.3389/fcell.2020.561804 PMC7591459

[65] Jia Q, Ding Q, Shao K, Dang J, Zhang F. Research progress regarding CYP3A gene family in gastric cancer. Zhong Nan Da Xue Xue Bao Yi Xue Ban. 2023;48(12):1874–81. 10.11817/j.issn.1672-7347.2023.230150. English, Chinese. 38448381 10.11817/j.issn.1672-7347.2023.230150 PMC10930750

[66] Zou J, Li H, Huang Q, Liu X, Qi X, Wang Y, Lu L, Liu Z. Dopamine-induced SULT1A3/4 promotes EMT and cancer stemness in hepatocellular carcinoma. Tumour Biol. 2017;39(10):1010428317719272. 10.1177/1010428317719272. 29025375 10.1177/1010428317719272

[67] Walker C, Mojares E, Del Río HA. Role of Extracellular Matrix in Development and Cancer Progression. Int J Mol Sci. 2018;19(10):3028. 10.3390/ijms19103028. 30287763 10.3390/ijms19103028 PMC6213383

[68] Gilkes DM, Semenza GL, Wirtz D. Hypoxia and the extracellular matrix: drivers of tumour metastasis. Nat Rev Cancer. 2014;14(6):430–9. 10.1038/nrc3726. 24827502 10.1038/nrc3726 PMC4283800

[69] Faes S, Dormond O. PI3K and AKT: Unfaithful Partners in Cancer. Int J Mol Sci. 2015;16(9):21138–52. 10.3390/ijms160921138. 26404259 10.3390/ijms160921138 PMC4613246

[70] Tan X, Yan Y, Song B, Zhu S, Mei Q, Wu K. Focal adhesion kinase: from biological functions to therapeutic strategies. Exp Hematol Oncol. 2023;12(1):83. 10.1186/s40164-023-00446-7. 37749625 10.1186/s40164-023-00446-7 PMC10519103

[71] Fu H, Zhu Y, Wang Y, Liu Z, Zhang J, Xie H, Fu Q, Dai B, Ye D, Xu J. Identification and Validation of Stromal Immunotype Predict Survival and Benefit from Adjuvant Chemotherapy in Patients with Muscle-Invasive Bladder Cancer. Clin Cancer Res. 2018;24(13):3069–78. 10.1158/1078-0432.CCR-17-2687. 29514839 10.1158/1078-0432.CCR-17-2687

[72] Takayama H, Nishimura K, Tsujimura A, Nakai Y, Nakayama M, Aozasa K, Okuyama A, Nonomura N. Increased infiltration of tumor associated macrophages is associated with poor prognosis of bladder carcinoma in situ after intravesical bacillus Calmette-Guerin instillation. J Urol. 2009;181(4):1894–900. 10.1016/j.juro.2008.11.090. 19237175 10.1016/j.juro.2008.11.090

[73] Hanada T, Nakagawa M, Emoto A, Nomura T, Nasu N, Nomura Y. Prognostic value of tumor-associated macrophage count in human bladder cancer. Int J Urol. 2000;7(7):263–9. 10.1046/j.1442-2042.2000.00190.x. 10910229 10.1046/j.1442-2042.2000.00190.x

[74] Takeuchi H, Tanaka M, Tanaka A, Tsunemi A, Yamamoto H. Predominance of M2-polarized macrophages in bladder cancer affects angiogenesis, tumor grade and invasiveness. Oncol Lett. 2016;11(5):3403–8. 10.3892/ol.2016.4392. 27123124 10.3892/ol.2016.4392 PMC4841030

[75] Naoe M, Ogawa Y, Takeshita K, Morita J, Iwamoto S, Miyazaki A, Yoshida H. Bacillus Calmette-Guérin-pulsed dendritic cells stimulate natural killer T cells and gammadeltaT cells. Int J Urol. 2007;14(6):532–8. 10.1111/j.1442-2042.2006.01697.x. discussion 538. 17593099 10.1111/j.1442-2042.2006.01697.x

[76] Joseph M, Enting D. Immune Responses in Bladder Cancer-Role of Immune Cell Populations, Prognostic Factors and Therapeutic Implications. Front Oncol. 2019;9:1270. 10.3389/fonc.2019.01270. 31824850 10.3389/fonc.2019.01270 PMC6879653

[77] Rosenblat M, Volkova N, Ward J, Aviram M. Paraoxonase 1 (PON1) inhibits monocyte-to-macrophage differentiation. Atherosclerosis. 2011;219(1):49–56. 10.1016/j.atherosclerosis.2011.06.054. 21798540 10.1016/j.atherosclerosis.2011.06.054

[78] Li XL, Liu YY, Knight D, Odaka Y, Mathis JM, Shi R, Glass J, Zhang QJ. Effect of B7.1 costimulation on T-cell based immunity against TAP-negative cancer can be facilitated by TAP1 expression. PLoS One. 2009;4(7):e6385. 10.1371/journal.pone.0006385. 19629186 10.1371/journal.pone.0006385 PMC2711302

[79] Van Kaer L, Ashton-Rickardt PG, Ploegh HL, Tonegawa S. TAP1 mutant mice are deficient in antigen presentation, surface class I molecules, and CD4-8+ T cells. Cell. 1992;71(7):1205–14. 10.1016/s0092-8674(05)80068-6. 1473153 10.1016/s0092-8674(05)80068-6

[80] Vonderheide RH, Glennie MJ. Agonistic CD40 antibodies and cancer therapy. Clin Cancer Res. 2013;19(5):1035–43. 10.1158/1078-0432.CCR-12-2064. 23460534 10.1158/1078-0432.CCR-12-2064 PMC3590838

[81] Beatty GL, Chiorean EG, Fishman MP, Saboury B, Teitelbaum UR, Sun W, Huhn RD, Song W, Li D, Sharp LL, Torigian DA, O’Dwyer PJ, Vonderheide RH. CD40 agonists alter tumor stroma and show efficacy against pancreatic carcinoma in mice and humans. Science. 2011;331(6024):1612–6. 10.1126/science.1198443. 21436454 10.1126/science.1198443 PMC3406187

[82] Zhu YD, Lu MY. Increased expression of TNFRSF14 indicates good prognosis and inhibits bladder cancer proliferation by promoting apoptosis. Mol Med Rep. 2018;18(3):3403–10. 10.3892/mmr.2018.9306. 30066919 10.3892/mmr.2018.9306

[83] Slebioda TJ, Rowley TF, Ferdinand JR, Willoughby JE, Buchan SL, Taraban VY, Al-Shamkhani A. Triggering of TNFRSF25 promotes CD8⁺ T-cell responses and anti-tumor immunity. Eur J Immunol. 2011;41(9):2606–11. 10.1002/eji.201141477. 21688261 10.1002/eji.201141477

[84] Zhao Y, Liu Z, Deng K, Qu H, Zhang Q, Zhou P, Yang M, Yang X, Wang H, Li R, Xia J. Identification of TAP1 as a T-cell related therapeutic target in gastric cancer by mediating oxalipliatin-related synergistic enhancement of immunotherapy. Int Immunopharmacol. 2024;132: 111998. 10.1016/j.intimp.2024. 38593510 10.1016/j.intimp.2024.111998

[85] Chen C, Yang Y, Guo Y, He J, Chen Z, Qiu S, Zhang Y, Ding H, Pan J, Pan Y. CYP1B1 inhibits ferroptosis and induces anti-PD-1 resistance by degrading ACSL4 in colorectal cancer. Cell Death Dis. 2023;14(4):271. 10.1038/s41419-023-05803-2. 37059712 10.1038/s41419-023-05803-2 PMC10104818

[86] Chabanon RM, Pedrero M, Lefebvre C, Marabelle A, Soria JC, Postel-Vinay S. Mutational Landscape and Sensitivity to Immune Checkpoint Blockers. Clin Cancer Res. 2016;22(17):4309–21. 10.1158/1078-0432.CCR-16-0903. 27390348 10.1158/1078-0432.CCR-16-0903

[87] Hollis PR, Mobley RJ, Bhuju J, Abell AN, Sutter CH, Sutter TR. CYP1B1 Augments the Mesenchymal, Claudin-Low, and Chemoresistant Phenotypes of Triple-Negative Breast Cancer Cells. Int J Mol Sci. 2022;23(17):9670. 10.3390/ijms23179670. 36077068 10.3390/ijms23179670 PMC9456208

[88] Yonezawa A, Masuda S, Yokoo S, Katsura T, Inui K. Cisplatin and oxaliplatin, but not carboplatin and nedaplatin, are substrates for human organic cation transporters (SLC22A1-3 and multidrug and toxin extrusion family). J Pharmacol Exp Ther. 2006;319(2):879–86. 10.1124/jpet.106.110346. 16914559 10.1124/jpet.106.110346

[89] Yokoo S, Masuda S, Yonezawa A, Terada T, Katsura T, Inui K. Significance of organic cation transporter 3 (SLC22A3) expression for the cytotoxic effect of oxaliplatin in colorectal cancer. Drug Metab Dispos. 2008;36(11):2299–306. 10.1124/dmd.108.023168. 18710896 10.1124/dmd.108.023168

[90] Filppula AM, Neuvonen PJ, Backman JT. In vitro assessment of time-dependent inhibitory effects on CYP2C8 and CYP3A activity by fourteen protein kinase inhibitors. Drug Metab Dispos. 2014;42(7):1202–9. 10.1124/dmd.114.057695. 24713129 10.1124/dmd.114.057695

[91] Okumoto K, Tamura S, Honsho M, Fujiki Y. Peroxisome: Metabolic Functions and Biogenesis. Adv Exp Med Biol. 2020;1299:3–17. 10.1007/978-3-030-60204-8_1. 33417203 10.1007/978-3-030-60204-8_1

[92] Kirtonia A, Sethi G, Garg M. The multifaceted role of reactive oxygen species in tumorigenesis. Cell Mol Life Sci. 2020;77(22):4459–83. 10.1007/s00018-020-03536-5. 32358622 10.1007/s00018-020-03536-5 PMC11105050

[93] Molla G, Bitew M. Revolutionizing Personalized Medicine: Synergy with Multi-Omics Data Generation, Main Hurdles, and Future Perspectives. Biomedicines. 2024;12(12):2750. 10.3390/biomedicines12122750. 39767657 10.3390/biomedicines12122750 PMC11673561

[94] DeGraff DJ. Novel use of a combined artificial intelligence approach to identify patients with noninvasive urothelial cell carcinoma of the urinary bladder who are at greatest risk for progression to muscle-invasive disease: a step forward. Eur Urol. 2010;57(3):407–8. 10.1016/j.eururo.2009.11.036. discussion 408–9. 19945780 10.1016/j.eururo.2009.11.036

[95] Khatami F, Hassanzad M, Nikfar S, Guitynavard F, Karimaee S, Tamehri Zadeh SS, Gholami K, Rezaeian A, Feiz-Abadi SA, Jahanshahi F, Aghamir SMK. The importance of personalized medicine in urological cancers. J Diabetes Metab Disord. 2021;21(1):841–52. 10.1007/s40200-021-00824-0. 35673449 10.1007/s40200-021-00824-0 PMC9167380

[96] D’souza AA, Tulpule V, Zang PD, Quinn DI. Bladder cancer: from a therapeutic wilderness to so many options; a guide to practice in a changing landscape. Ann Oncol. 2022;33(3):242–3. 10.1016/j.annonc.2022.01.073. 35151428 10.1016/j.annonc.2022.01.073

[97] Audisio M, Buttigliero C, Turco F, Delcuratolo MD, Pisano C, Parlagreco E, Di Stefano RF, Di Prima L, Crespi V, Farinea G, Cani M, Tucci M. Metastatic Urothelial Carcinoma: Have We Take the Road to the Personalized Medicine? Cells. 2022;11(10):1614. 10.3390/cells11101614. 35626651 10.3390/cells11101614 PMC9139766

[98] Bergerot CD, Schmitz KH, Crane TE, Klaassen Z, Bergerot PG. Comprehensive care for patients with bladder cancer: Addressing the interplay of physical, nutritional, and emotional well-being. Cancer. 2025;131(1): e35670. 10.1002/cncr.35670. 39607007 10.1002/cncr.35670

[99] Rutten VC, Al CM, Festen S, Zuiverloon TCM, Boormans JL, Polinder-Bos HA. Selecting the right treatment: Health outcome priorities in older patients with bladder cancer. J Geriatr Oncol. 2024;15(6): 101811. 10.1016/j.jgo.2024.10181. 38896950 10.1016/j.jgo.2024.101811

[100] Liu H, Guo Z, Wang P. Genetic expression in cancer research: Challenges and complexity. Gene Reports. 2024: 102042. 10.1016/j.genrep.2024.102042.

[101] Liu H, Li Y, Karsidag M, Tu T, Wang P. Technical and Biological Biases in Bulk Transcriptomic Data Mining for Cancer Research. J Cancer. 2025;16(1):34–43. 10.7150/jca.100922. 39744578 10.7150/jca.100922 PMC11660120

