# Supplementary figures and images for: ADME gene-driven prognostic model for bladder cancer: a breakthrough in predicting survival and personalized treatment
Source: Hereditas. 2025 Mar 19;162:42. doi: 10.1186/s41065-025-00409-4 (PMC11921678; doi:10.1186/s41065-025-00409-4)

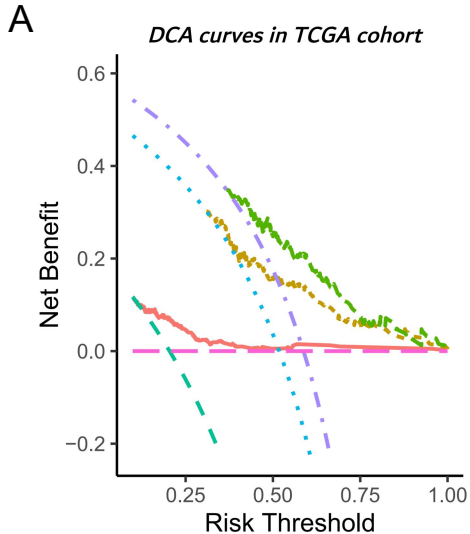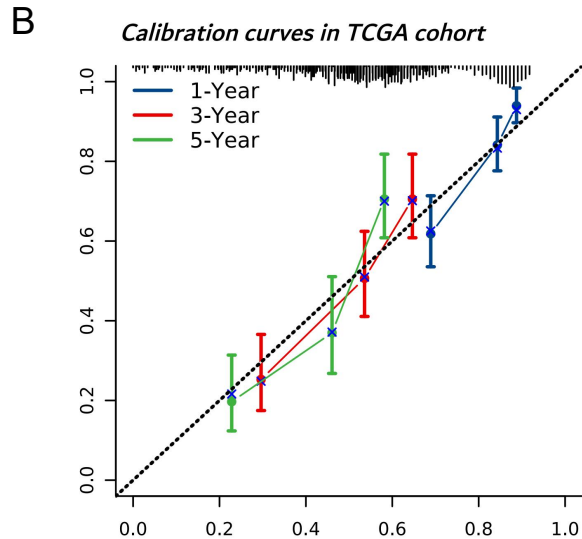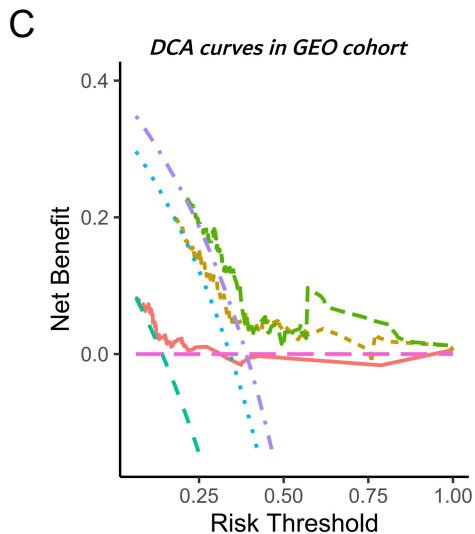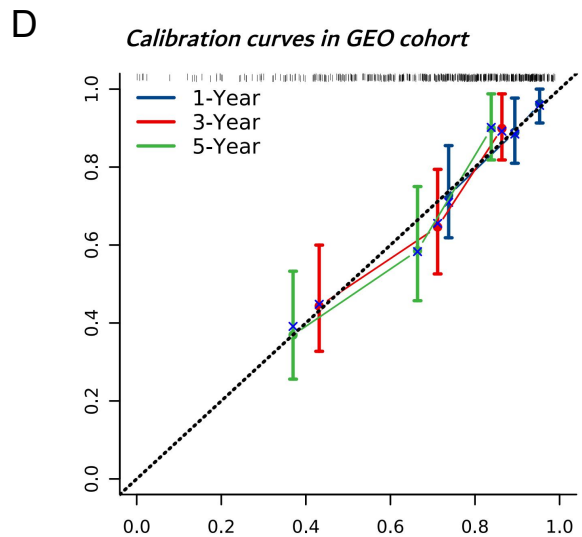

Supplement: Supplementary file 1 — Supplementary Material 1. [file 41065_2025_409_MOESM1_ESM.pdf]

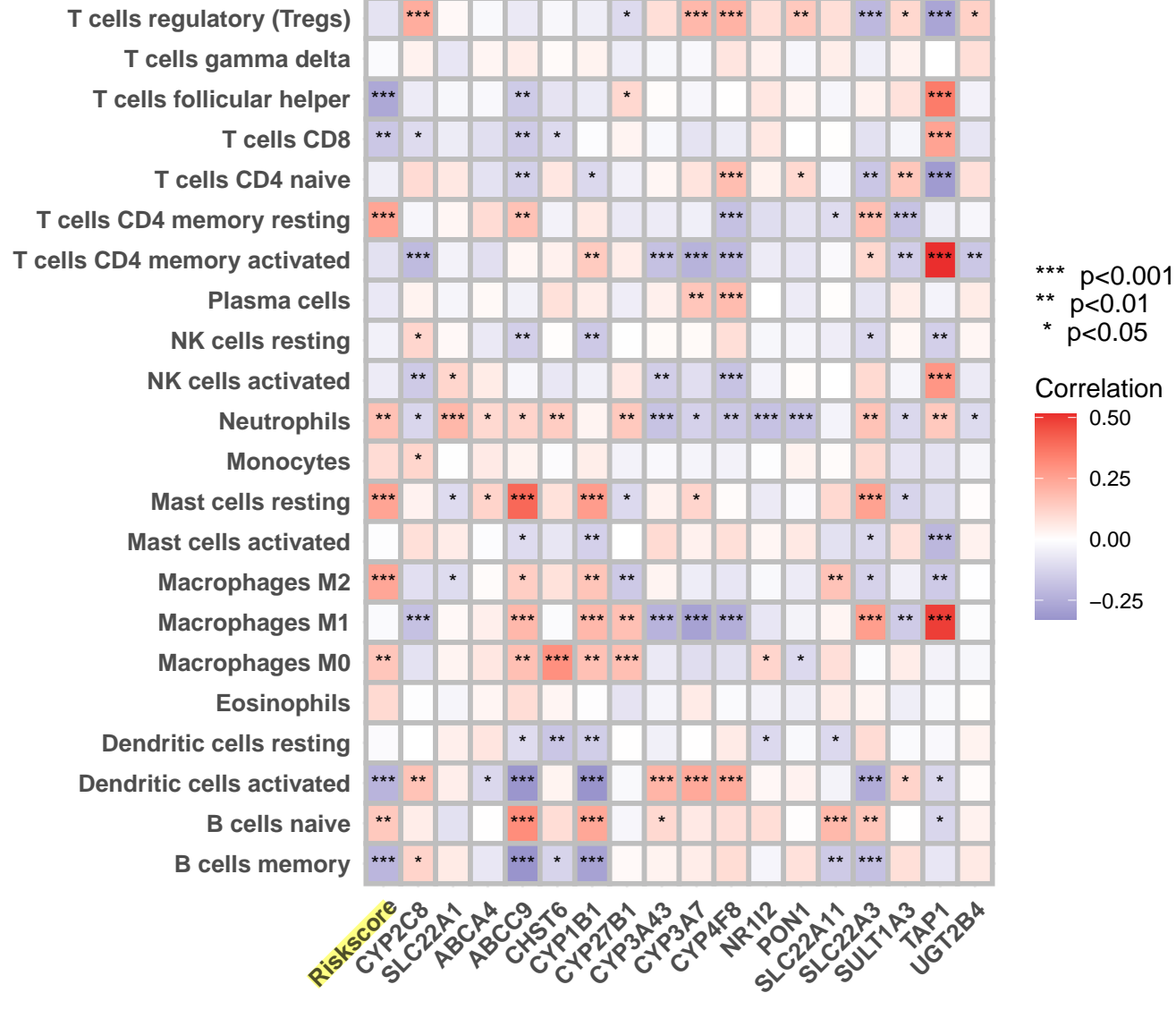

Supplement: Supplementary file 2 — Supplementary Material 2. [file 41065_2025_409_MOESM2_ESM.pdf]

A

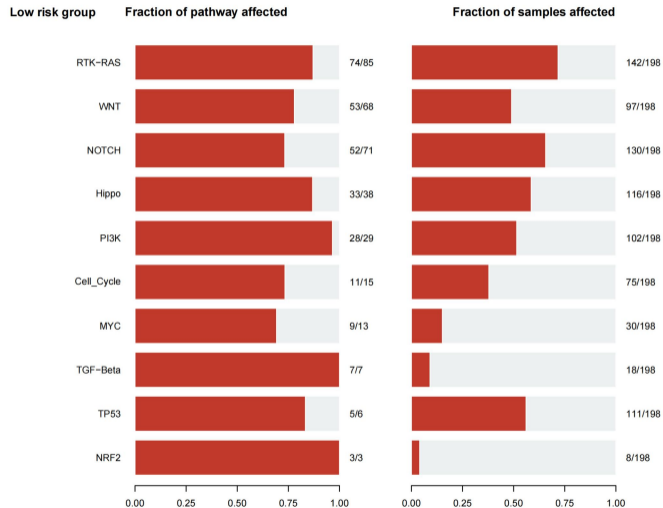

B

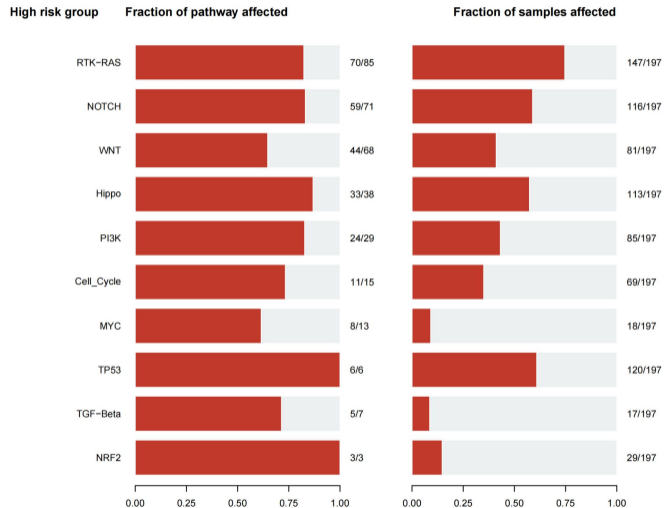

Supplement: Supplementary file 3 — Supplementary Material 3. [file 41065_2025_409_MOESM3_ESM.pdf]
